# Perspectives of individuals on reducing meat consumption to mitigate climate change – a scoping review

**DOI:** 10.1186/s40795-025-01171-6

**Published:** 2025-10-13

**Authors:** Ramona Moosburger, Almut Richter, Gert B. M. Mensink, Kristin Manz, Julia Wagner, Katharina Heldt, Julika Loss

**Affiliations:** 1 Epidemiology and Health Monitoring, Robert Koch Institute, Berlin, Germany; 2 Method Development, Research Infrastructure and Information Technology, Robert Koch Institute, Berlin, Germany

**Keywords:** Climate protection, Reducing meat consumption, Mitigation, Awareness, Willingness, Motivation, Systematic review

## Abstract

**Background:**

As high meat consumption is detrimental to both individual health and the climate, many international organizations recommend a reduction in meat consumption among populations. This scoping review aims to synthesize the evidence on individuals' perspectives on reducing meat consumption to mitigate climate change. The three research questions focus on (1) the individuals' awareness of the link between meat consumption and climate change, (2) individuals' willingness to reduce their meat consumption to mitigate climate change, and (3) individuals who have already reduced their meat consumption for this purpose.

**Methods:**

This scoping review follows the extended PRISMA guidelines for scoping reviews. A systematic search was conducted in five databases (Medline, Scopus, Embase, Greenfile and PsynDex/CurrentContent/Agris via Livivo). Only peer-reviewed original studies, published since 2015, in English, German, Danish or Dutch were included. Two researchers performed all screening procedures. Data from included studies were summarized in a narrative and descriptive synthesis of evidence, separately for quantitative and qualitative studies.

**Results:**

A total of 93 studies were included. The majority of studies were published since 2019, had a quantitative study design, and were conducted in Europe. Awareness of the link between meat consumption and climate change is low in most studies, and many people underestimate the climate change mitigation potential of meat consumption. Women and people with lower current meat consumption are more willing to reduce their meat consumption. Health and animal welfare are often the main reasons for reducing meat consumption, with climate change being a secondary motivation for most. However, studies varied in the questionnaires used, and many had small sample sizes, limiting comparability and generalizability.

**Conclusions:**

Further research using nationwide samples and standardized, validated instruments would improve insight into individuals’ perspectives on reducing meat consumption to mitigate climate change and is crucial to understanding of how to effectively promote a more plant-based diet. As media and political attention to climate change mitigation is increasing, it will be valuable to monitor changes in individual awareness, willingness and motivation to reduce meat consumption across populations worldwide.

**Trial registration:**

This scoping review has been registered at Open Science Framework (https://doi.org/10.17605/OSF.IO/MWB85) and the review protocol has been published in BMJ Open.

## Background

Climate change is recognized as one of the greatest public health threats of the twenty-first century [1]. The consequences of climate change have a direct impact on human health, well-being and security. The frequency of life-threatening weather events, such as droughts and heat waves, will continue to increase, leading to, among other things, increased heat-related mortality, accidents and trauma. Rising average temperatures are already having an impact on ecosystems, causing an increase in certain infectious diseases and allergies. We must therefore proactively reduce our greenhouse gas emissions to mitigate climate change. In the 2015 Paris Agreement, many countries committed to limiting global warming to a maximum of 1.5 degrees Celsius above pre-industrial levels [2]. However, the transformations needed across all sectors to achieve this goal appear to be happening too slowly [3].

The food system is responsible for 19%–29% of global greenhouse gas emissions, of which 80%–86% come from agricultural production [4]. Within agricultural production, meat production is responsible for 72%–78% of greenhouse gas emissions [5], with beef production emitting significantly higher amounts of greenhouse gases per unit of mass, per gram of protein, and per serving compared to pork and poultry production [6, 7]. As the world’s population and wealth continue to grow, there is a risk that the demand for meat and the negative environmental and climate impacts associated with meat production will continue to increase [5].

Diets high in meat, especially red and processed meat, are also associated with several chronic noncommunicable diseases, such as coronary heart disease, cancer, and type 2 diabetes, compared with diets low in meat [7–10]. In a meta-analysis, consumption of processed meat consumption was associated with a 42% higher risk of coronary heart disease and a 19% higher risk of diabetes [8]. Comparing meat eaters and vegetarians, the EPIC Oxford study found that vegetarians had a 10% lower risk of total cancer than meat eaters [11].

Due to the harmful effects that high meat consumption can have on both individual health and the climate, many international associations and expert commissions emphasize the need to reduce meat consumption in people’s diets. The EAT-Lancet Commission, for example, describes diet as the ‘single strongest lever to optimize human health and environmental sustainability on Earth’ [12], and recommends significant reductions in meat consumption, particularly in high-income countries. Similarly, the Food and Agriculture Organization of the United Nations and the World Health Organization support reducing meat consumption as an important step towards more sustainable and healthier diets [13]. On the other side, the meat industry tries to counteract these developments and defends its interests through lobbying, greenwashing attempts, and insistence that meat consumption is necessary for human health [14, 15].

Research has already identified typical individual barriers to meat reduction, including habits, taste preferences, or emotional attachments to meat [16, 17]. On the other hand, health and animal welfare concerns have been shown to be important reasons for individuals to reduce or stop eating meat [18–23]. Other motives may be related to the price of meat, food scandals (e.g., products containing horsemeat without appropriate declaration, contaminated meat products, or scandalous reports about conditions in factory farming), and social pressure from peers or family.

Less is known about the willingness of individuals to reduce their meat consumption in order to mitigate climate change. Some people may simply be unaware of or underestimate the climate impact of meat consumption and therefore be unwilling to change their diet. Awareness refers to an individual's conscious recognition or understanding of a health- or environment-related issue. This includes knowledge of risk factors, behaviors, potential impacts, and preventive measures**.** It is a cognitive state that plays a fundamental role in health behavior change [24]. Research on the reasons for meat avoidance and the willingness to change meat consumption is still scarce [25]. In health psychological theories, willingness refers to an individual's openness or readiness to engage in a particular health-related behavior, often (but not necessarily) without prior intention or planning [26]. It would be helpful to know which factors are most conducive for behavior change in order to design more effective prevention measures. Few studies have examined the motivations of meat reducers or flexitarians to reduce their meat consumption [27], and also research on the transition from meat to plant-based diets remains scattered [28]. Systematic reviews have found a generally low consumer awareness of the environmental impact of meat production and a low willingness to change meat consumption, and only a minor role for climate change in the decision to reduce meat consumption [27, 29, 30]. As these reviews were conducted in 2016 and 2018, they may not reflect the current situation, as global warming concerns have increased worldwide in recent years [31], and people may have become more aware of the drivers of climate change, for example as a result of the publication of the Planetary Health Diet by the EAT Lancet Commission in 2019 [32], and of new climate movements such as Fridays for Future [31], which started in 2018.

A better overview of individuals’ awareness, willingness, and motivations to reduce meat consumption for climate protection could be useful when conceptualizing health promotion campaigns.

Our scoping review summarizes the evidence on individuals’ perspectives on reducing their own meat consumption to mitigate climate change, and it structures the evidence on this topic according to three research questions (RQ):

- RQ1: **What do we know about individuals’ awareness of the link between their own meat consumption and the potential to mitigate climate change?**
- RQ2: **What is the current state of knowledge regarding individuals’ willingness to reduce meat consumption in order to mitigate climate change?**
- RQ3: **What is the current evidence on individuals who have reduced their meat consumption for climate change mitigation reasons?**

## Methods

Our scoping review follows the Preferred Reporting Items for Systematic reviews and Meta-Analyses extension for Scoping Reviews (PRISMA-ScR) [33]. The scoping review was registered at Open Science Framework (10.17605/OSF.IO/MWB85) and the intended methodology was published as part of a peer-reviewed protocol in BMJ Open [34].

### Search strategy

We systematically searched the databases Medline (via PubMed), Scopus, Embase, Greenfile (via Ebsco) and PsynDex/CurrentContent/Agris (via Livivo) on October 7, 2022. We updated the search twice; on October 2, 2023 and February 21, 2025. The detailed search strategy for each database, including each search string, is documented in our review protocol [34].

### Inclusion criteria

We included only original, peer-reviewed articles reporting one of the following study designs:

- Observational studies (longitudinal and cross-sectional studies),
- Qualitative studies,
- Intervention studies (we only extracted data from the baseline survey before the intervention or from the control group),
- Mixed methods studies (we only extracted data from those study components that met the inclusion criteria), and
- Reviews.

For reviews, we did not include the evidence from the review as such, but we screened the cited original articles and included them if they met the inclusion criteria.

Conference abstracts and other grey literature were not included.

In terms of populations, we included studies of meat eaters, so-called ‘flexitarians’, and vegetarians and vegans. We included studies in all age groups.

We considered the content of:

- Studies that examine individuals’ awareness of the link between their meat consumption and climate chance mitigation.
- Studies that address individuals’ willingness to reduce meat consumption.
- Studies investigating climate change mitigation as a motive for individuals who have already reduced their meat consumption.

We considered articles published in the languages English, German, Danish and Dutch. As we wanted to focus on recent evidence and to provide an update after previously published reviews on the topic [27, 29, 30], we included studies published between January 1, 2015 and February 21, 2025.

### Study selection

Two researchers (RM, JW/JR) performed the screening process. Twenty abstracts were screened together to ensure a common understanding of the inclusion and exclusion criteria. The remaining abstracts were screened independently by both. An article was considered for full-text screening if at least one researcher suggested an inclusion. Afterwards, the full-texts of the selected articles were reviewed. Initially, five articles were screened jointly by the two researchers together to harmonize the understanding of the inclusion and exclusion procedure. The remaining articles selected for full-text screening were again screened independently by both researchers. Disagreements during full-text screening (*n* = 11 of 128 full-texts screened in the original search) were discussed with and resolved by a third person (AR) from the review team. All included studies were manually searched for other relevant references (reference tracking).

### Data extraction

From articles meeting all inclusion criteria, details were extracted on study characteristics (authors, title, publication year, study design, country, study period); study methods (research question(s), sample size, sampling method); population characteristics (target population under study, percentage female, percentage vegetarians); measurements (of awareness, willingness, [change in] behavior and motives, meat type under study); key findings and author reported limitations. Two researchers (RM, JW) pilot tested the data extraction instrument with three articles, before one (RM) extracted the data from all included studies. A large sample (around 50%, *n* = 43 articles) was double checked (JW) and the other researchers of the review team were consulted whenever there was uncertainty to reach consensus.

### Data analysis

To answer the three research questions, the data from the included studies were summarized in a narrative and descriptive synthesis of the evidence. Methodological findings were synthesized according to the study design (quantitative or qualitative), and the content findings were synthesized for each research question. For this, results from quantitative studies, including baseline data from intervention studies, were synthesized first, followed by complementary evidence from qualitative studies. For one mixed method study, only the experimental part of the study (quantitative pretest data) and for a second mixed method study only qualitative data were extracted. Quantitative and qualitative parts of mixed methods studies were analyzed and categorized separately.

In alignment with the PRISMA-ScR extensions for scoping reviews, we did not perform a risk of bias assessment for each included study.

## Results

**Fig. 1:**
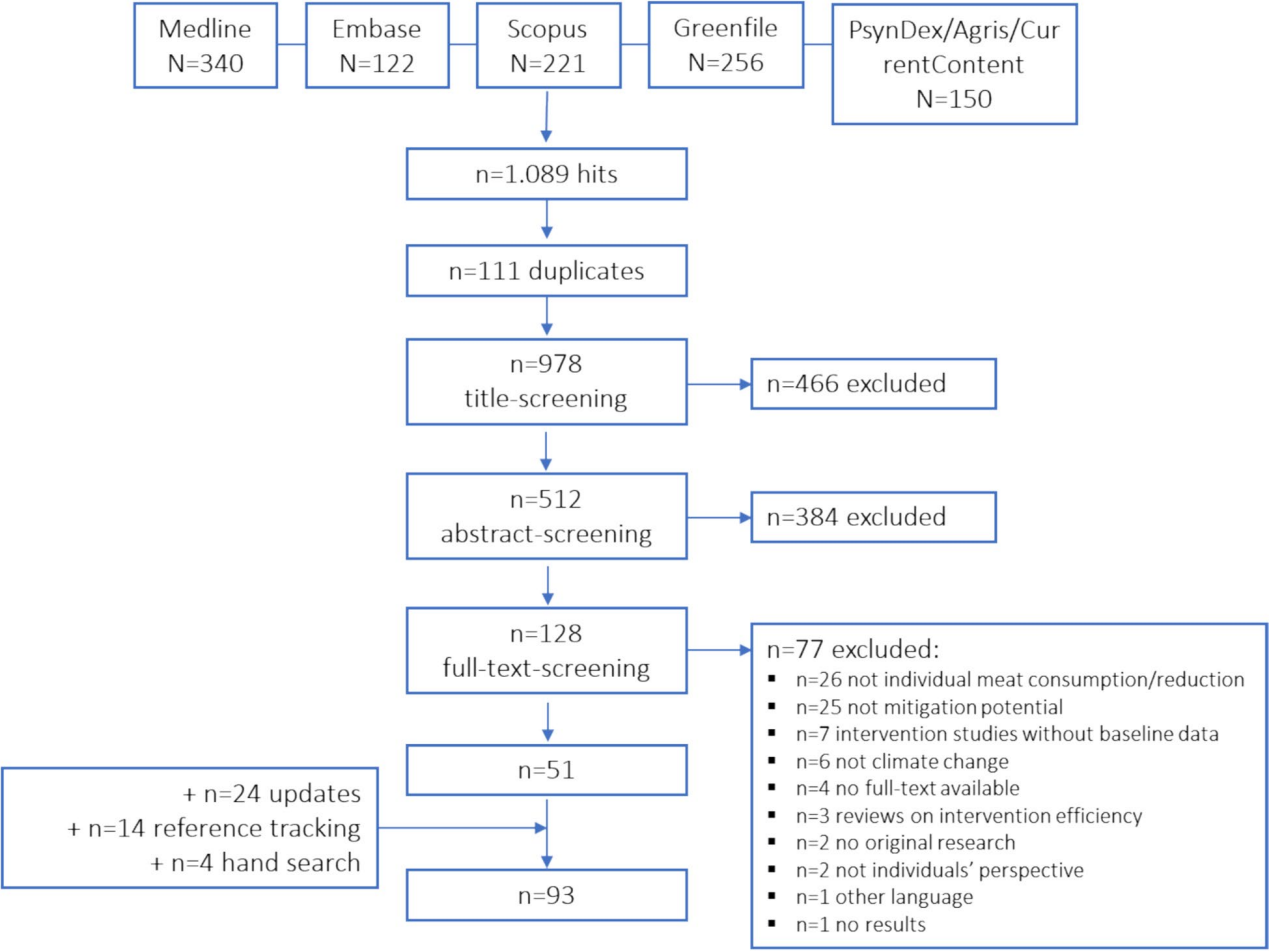
Flowchart

The original search in the databases identified 1,089 articles. 111 duplicates were removed. After title/abstract screening, 128 full-texts were reviewed. Of these, 51 met the inclusion criteria and 77 were excluded. In the first and second update twelve additional studies each, were found. Fourteen studies were included through reference tracking and four articles were found through hand searching, resulting in a total of 93 studies included in this scoping review (see flowchart in Fig. 1).

**Fig. 2:**
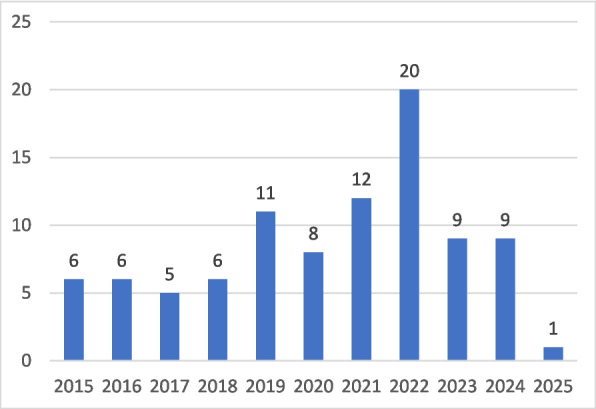
Articles included (n) in the scoping review by publication year

Of the included articles, 57 were quantitative studies [20, 22, 35–89], 20 were qualitative studies [18, 21, 90–107], five were intervention studies with a quantitative baseline survey or control group [108–112], five were mixed method studies [113–117], and six were reviews [16, 25, 27–30]. The majority of the studies were published in the year 2019 or later (Fig. 2).

**Fig. 3:**
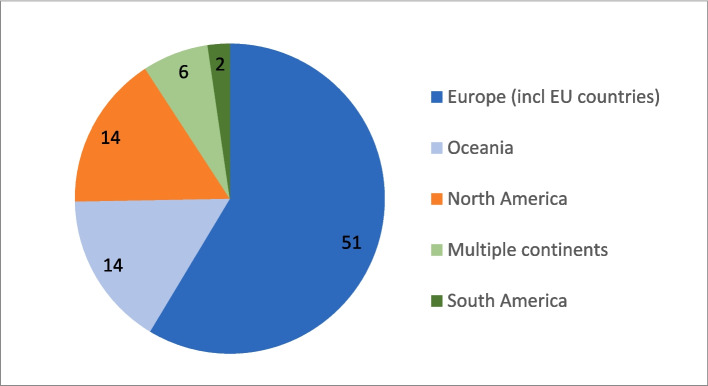
Studies included (n) in the scoping review by world regions (excluding *n* = 6 reviews)

The majority of studies were conducted in Europe (*n* = 51), followed by Oceania and North America (Fig. 3).

### Methodology-related findings

#### Quantitative studies

Fifty-seven observational studies [20, 22, 35–89], five intervention studies [108–112] and four mixed method studies [113–115, 117] are included in this overview. This makes a total of 66 studies.

Almost all quantitative studies were cross-sectional, with only one exception being a longitudinal study [78]. The sample sizes range from 60 to 27,237 participants, the percentage of women from 37% to 80.3%, and the percentage of vegetarians (including vegans) included in the study range from 0 to 100%. Most studies examine awareness, willingness, and motives for meat reduction in the adult population. Table 1 shows the sampling strategies used. Twenty studies claim to be representative of a national population [36, 45, 46, 51, 54, 55, 57, 61, 64, 66, 67, 71, 77, 81, 84–87, 109, 112].

**Table 1:** Sampling strategies used in the included quantitative and qualitative studies

Sampling strategy	Quantitative studies	Qualitative studies
Panel with quota	13	1
Panel without quota	13	1
Convenience or voluntary response sampling	13	16
Randomly selected	9	-
Snowball sampling	1	-
Quota sampling	1	-
Other or more than one strategy of sampling	12	4
No specification	4	2

In the majority of studies, the authors did not specifically define “meat”, while one study only excluded fish [47] and one study specifically included chicken [22]. Eight studies focus explicitly on red meat (some also include processed meat in their definition) [36, 39, 53, 68, 74, 80, 88, 112], six studies combine different types of red and white meat [46, 49, 54, 63, 75, 115], and seven also include fish in their definition of meat [55, 57, 61, 64, 67, 73, 111].

Author reported study limitations of the quantitative studies were related to sample characteristics (e.g., overrepresentation of highly educated people and women), sampling methods, use of self-reported data and associated social desirability bias, limited generalizability, and lack of longitudinal study design and thus the ability to examine behavior change over time.

#### Qualitative studies

Twenty qualitative [18, 21, 90–107] and four mixed method studies [113–116] are included in this overview. This makes a total of 24 studies.

Most of the qualitative studies conducted focus groups (*n* = 11) [18, 21, 90, 91, 93, 100, 102, 103, 105, 113, 114], nine studies used individual interviews [92, 94–96, 99, 104, 106, 107, 115], one study conducted both [98] and three studies used open-ended questions in an inductive qualitative approach [97, 101, 116]. The number of participants ranged from 13 to 701, the percentage of women from 32 to 87%, and the percentage of vegetarians from 0 to 67%. The most common target population studied was adults. The majority of the qualitative studies used convenience or voluntary response sampling strategies (Table 1).

In the vast majority of studies, meat was not defined by the authors (*n* = 21), however the participants in one study were asked to define meat for themselves: They generally agreed that meat included red, white, and processed animal flesh [100]. One study focussed on red meat [97] and two studies considered different types of red and white meat [93, 115].

In the qualitative studies, the main limitations cited by the authors were small sample sizes, non-probabilistic sampling methods and, consequently, lack of generalizability and selection bias.

### Content-related findings

#### Research question 1: What do we know about individuals’ awareness of the link between their own meat consumption and the potential to mitigate climate change?

Fifty-four of the included articles provide results relevant to the first research question, including 33 quantitative studies [36–41, 43, 44, 50–53, 55, 57, 59–62, 64–66, 68, 69, 71, 72, 75–81, 89], 17 qualitative studies [18, 90–95, 97–100, 102–107], two intervention study [109, 112] and two mixed method studies [113, 114] (see Table 2).

**Table 2:** Key findings of included studies

**Quantitative studies**								
**Author, publication year**	**Country, Study design, Population under study**	**Sampling method, sample size**	**Indicator measured** **RQ1: Awareness**	**Key findings** **RQ1: Awareness**	**Indicator measured** **RQ2: Willingness to reduce**	**Key findings** **RQ2: Willingness to reduce**	**Indicator measured** **RQ3: Motives for reduction**	**Key findings** **RQ3: Motives for reduction**
** Arnaudova et al., 2022** [35]	Switzerland Cross-sectional Students	Multiple sampling methods (through universities, flyers, student groups on facebook) *N* = 498			Transtheoretical model (4 stages of change)	14.7% precontemplation, 6% contemplation, 44.7% preparation, 34.6% action/maintenance stage		
** Aureli et al., 2023** [36]	Italy Cross-sectional Adults	Panel incl. quota (gender, age, area of residence) Representative *N* = 815	'To what extent do the following activities contribute to climate change?' Scale 1–10	56% agree that the production of meat and dairy products, which we eat and drink, contribute to climate change (7–10 points)	'I am willing to cut down on red meat (beef, pork, lamb).' 10-point scale ‘I’ve reduced the consumption of red meat (but still it it)’; ‘I’m intending to reduce the consumption of red meat for environmental reasons’; ‘I’ve stopped eating red meat for environmental reasons (though I’m not vegetarian/vegan)’; ‘I’m intending to stop eating red meat for environmental reasons’ yes or no	57% are willing to cut down on red meat (7–10 points on scale) 10% intend to reduce red meat consumption for environmental reasons 1% intend to stop eating red meat for environmental reasons 51% have already reduced their consumption of meat for environmental reasons Older people were more likely to have reduced than younger people 7% have stopped eating red meat for environmental reasons (but are not vegetarian)	‘Have you reduced (or plans to reduce) the consumption of red meat (beef, lamb and pork) for environmental reasons?’ Yes or no	51% have already reduced their consumption of red meat for environmental reasons Older people were more likely to have reduced than younger people 7% have stopped eating red meat for environmental reasons (but are not vegetarian)
** Austgulen et al., 2018** [113]	Norway Mixed-method Adults	Panel *N* = 1,532	‘Production, distribution and consumption of food is connected with significant environmental impact. Which of the following measures do you think will have the greatest impact on the environment?’ reduce food waste, increase production and consumption of local food, increase production and consumption of organic food, reduce production and consumption of meat Scale 1–4	Consumers tended to underestimate the environmental impact of meat consumption. Only 33% ranked reducing meat consumption as beneficial (15% score 1 and 18% score 2) Norwegian consumers rank purchasing organic food (mean = 3.1) and foregoing meat (mean = 2.9) as the least environmentally beneficial. Increasing the production of locally produced food (mean = 2.0) and reducing food waste (mean = 2.0) are ranked as the most environmentally beneficial measures Furthermore, almost a third of the respondents answered that they did not know or that neither of the alternatives were environmentally beneficial			'Have environmental problems caused you to reduce your meat consumption?' yes or no	Only 14% of the respondents claim to actually have reduced their consumption of meat due to environmental reasons
** Banos-González et al., 2021** [108]	Spain Experimental study design Students	- *N* = 144			Willingness to take personal actions related to food consumption which could help to offset climate change were assessed: pay more to buy local products, give up certain products which are not in season, eat less meat 4-point Likert scale	In the pretest, both groups were more willing to pay more to buy local products or to give up certain products which are not in season, than to reduce their meat consumption		
** Bogueva & Marinova, 2022** [37]	Australia Cross-sectional Young adults (Gen Z)	Panel *N* = 478	Participants had to choose from a list of options for the factors they considered to have a major influence on climate change They were also asked directly whether people’s food choices are a major contributor to climate change (yes or no)	38% believe that livestock production and the consumption of animal-sourced foods are a major contributor to climate change. (chosen from a list) When directly asked (yes or no), 34% agreed that food choices are a major contributor to climate change. 66% disagreed				
** Bryant, 2019** [38]	United Kingdom Cross-sectional Adults (meat eater)	Convenience *N* = 1,000	Asked to give their opinions about 11 different aspects of vegetarian and vegan diets – > ‘good for environment’ 7-point Likert scale	A large majority (74% and 69%) of UK meat-eaters said that vegetarian and vegan diets are good for the environment. (5–7 points) mean: approx. 5.4	Questions about their intended consumption of meat one month from today 6-point scale: eliminate, greatly reduce, slightly reduce, maintain about the same, slightly increase or greatly increase	0.1% eliminate, 3.5% greatly decrease, 13.0% slightly decrease – > 16.6% intend to decrease meat consumption one month from today 81.0% maintain the same, 1.9% slightly increase, 0.5% greatly increase Mean = 3.8 Higher education was correlated with increased likelihood to say they would reduce their consumption of meat		
** Campbell-Arvai, 2015** [114]	United States of America Mixed-methods Students	Convenience (dining hall) *N* = 320	‘Eating less meat can help the environment.’ ‘Adopting a vegetarian diet can help the environment.’ 5-point Likert scale	28.7% agreed with the statement that eating less meat could help the environment (score 4–5) 21.6% agreed that a vegetarian diet could benefit the environment (score 4–5) Over 50% indicated disagreement with the connection between reduced meat consumption and environmental benefits Most students were in strong agreement that recycling (food and drink containers) and reuse (food containers and drink bottles) can best help the environment Reducing meat consumption and vegetarian diet were ranked least effective				
** Clonan et al., 2015** [39]	United Kingdom Cross-sectional Adults (from Nottinghamshire area)	Randomly selected *N* = 842	‘To help reduce the impact of climate change, it is better to eat less animal foods (meat, dairy products and eggs)’ 5-point Likert scale	18·4% agreed or agreed strongly that the impact of climate change could be reduced by consuming less meat, dairy products and eggs (4 or 5 points). 36% disagreed or disagreed strongly (1 or 2 points) No significant difference between gender, age group and socioeconomic group				
** Çoker et al., 2024** [40]	United Kingdom Cross-sectional adults	Panel with quota *N* = 1,014	“Eating less red and processed meat is better for the environment” 5-point Likert scale	27–35% strongly agree 32–35% tent to agree 15–23% don't know 9–12% tend to disagree 5–8% strongly disagree The majority of the population (64.3%) agreed that overconsumption of red and processed meat has a negative impact on the environment. No statistical significant difference between White respondents, South Asian and Black respondents Women had a higher likelihood of believing meat consumption had negative impacts on the environment than men (OR = 1.55) Lower-SEP respondents were less likely to believe that overconsumption of red and processed meat was bad for the environment compared their higher-SEP counterparts in the total sample (OR = 0.63)	“Are you considering decreasing or increasing the amount of meat you eat?” recently decreased, wants to decrease, no change (non-meat eater), no change, wants to incease, recently increased	23–28% recently decreased 11–19% wants to decrease 7–12% no change (non-meat eaters) 30–52% no change (meat-eater) South Asian participants were more likely than White respondents to want to decrease or to have recently decreased their meat consumption (OR = 1.82) There were no statistically significant differences between men and women in the overall sample in wanting to decrease meat consumption, but White women were more likely to want to decrease or to have recently decreased their meat intake than their male counterparts (OR = 1.79) In the overall sample, there were no statistically significant differences between lower-SEP and higher-SEP respondents in their likelihood of decreasing their meat consumption		
**de Boer & Aiking, 2017 **[42]	27 EU countries Cross-sectional EU citizens	Randomised probability sampling			‘Some people say large scale meat production has a negative impact on the environment. Would you be willing to do the following for environmental reasons …?: Replace most of the meat you eat by vegetables’ Yes or no	In total 51% were willing to replace meat with vegetables Replacing most of the meat by vegetables was responded to more positively in the two Mediterranean zones (55% and 57%) than in the Northern zone (38%) The percentage of positive responses, ranging between 70% (Romania) and 29% (the Netherlands), strongly decreased along the south-north gradient and to a lesser degree, with higher levels of GDP Willingness to replace meat with vegetables had a strong correlation with frequency of meat consumption (willingness higher when less meat consumption) and gender (women more willing)		
** de Boer et al., 2016** [41]	United States of America, The Netherlands Cross-sectional Adults	Quota sampling N(USA) = 556 N(NL) = 527	‘For each of the following lifestyle-changes, please let us know whether you think this is an effective way of combatting climate change’: Eat less meat, buy local/seasonal/unprocessed foods (e.g., by going to farmer's markets), buy (more) organic foods 5-point Likert scale	‘Eating less meat’ was the least effective (USA) or the least effective but one (NL) option in the eyes of the participants	‘For each of the following lifestyle-changes (eat less meat, buy local, seasonal, unprocessed foods, buy organic foods), please let us know whether you are willing to personally make that lifestyle change (if you are already doing it, you are willing).’ 5-point Likert scale	Dutch sample M = 3.6, American sample M = 3.0 The willingness to eat less meat increased with the option's perceived effectiveness (in the Netherlands exclusively), not being a regular meat eater, and female gender In both samples lowest willingness to eat less meat compared to other lifestyle changes		
** de Boer et al., 2017** [22]	The Netherlands Cross-sectional Young adults (native Dutch and second generation Chinese Dutch)	Quota sampling (They were addressed in the street or contacted at home or through clubs, societies and eating places) *N* = 357 Native Dutch *N* = 350 Chinese Dutch					Asked to indicate a maximum of three reasons for not frequently eating meat (9 options): Because it's better for the environment, animal welfare, I like to vary, health, cost, that’s what I am used to, religion, other people in my household, don’t like meat	“It is better for the environment” was mentioned by vegetarians, low and middle meat eaters (20–40%), but was not specific for a particular dietary group The vegetarians reported two key reasons, namely “I don't like meat very much” (71% (natives), 47% (Chinese)) and “I think animal welfare is important” (71% (natives), 68% (Chinese)) Unlike the vegetarians, about 30%−40% of the non-vegetarians referred to financial reasons for not frequently eating meat The low and medium meat-eaters often considered health a reason to eat meat as well as to moderate meat eating, plus they liked to vary their meals. In these aspects they were different from both the vegetarians and the high meat-eaters
** de Boer et al., 2022** [43]	27 EU countries Cross-sectional EU citizens	Randomly selected *N* = 10,569 (Northwest countries) *N* = 16,668 (East and South countries)	‘We often hear people talking about the importance of eating a healthy and sustainable diet. What do you think ‘eating a healthy and sustainable diet’ involves?' 15 answer categories (Two meat-related: 'Eating meat less often’; ‘Eating vegetarian or vegan’)	The most popular items were ‘Eating a variety of different foods, having a balanced diet’ and ‘Eating more fruits and vegetables.’ The item ‘Eating meat less often’ was in the middle between the more popular and the less popular items, slightly higher (7th; 38%) in the NW region and lower (12th; 27%) in the E & S region. ‘Eating vegetarian or vegan’ was the least chosen item				
** de Gavelle et al., 2019** [44]	France Cross-sectional Adults	Panel incl. quota *N* = 2,055	‘To help reduce the impact of climate change, it is better to eat less animal foods (meat, dairy products and eggs)’ ‘Substituting beans for meat slows down climate change.’ 7-point Likert scale	Compared with omnivores (approx. 3.5), pro-flexitarians (approx. 5.0), flexitarians (approx. 5.5) and vegetarians (approx. 6.0) were much more in agreement about the mitigation potential of consuming less meat, dairy products and eggs	‘I intend to reduce my meat consumption in the coming months.’ ‘I am considering eating meat and fish only very rarely (no more than once a week).’ 7-point Likert scale Theory of planned behavior	The most marked difference (> 30%) between pro-flexitarians and omnivores were the estimates of intent (INT + 62%) to reduce meat intake Omnivore M = approx. 3.0, Pro-Flexitarian M = approx. 5.0, Flexitarian M = approx. 5.5 (estimated percentages out of a figure) Attitudes, subjective norms and perceived behavioral control explained 51% of the variance of intention		
** Dillon-Murray et al., 2024** [45]	Australia Cross-sectional adults	Survey platform Representative *N* = 492			“Please indicate your willingness to reduce your consumption of animal products” 5-point Likert scale and option “not applicable, already vegan”	49% willing to reduce (score 4–5), 27% not willing (score 1–2) 28.2% of the variance in willingness was explained by introducing motivations Health motivation predicted a lower level of willingness to reduce meat consumption (β = −0.14 p <.001), whilst environmental (β = 0.51, p <.001) and animal motivations (β = 0.16, p <.002 predicted a higher willingness to reduce meat consumption Environmental motivation was the best predictor of willingness to reduce consumption, accounting for 51% of the variance Empathy was not associated with willingness to reduce animal product consumption	The Vegetarian Eating Motives Inventory (VEMI) has 15 items with 7-point Likert Scales with three subscales (Animal, Environment, and Health); each has five items Example Environment: "Eating meat is bad for the planet"	Health M = 5.6, Animal M = 5.2, Environment M = 4.5
** Downs et al., 2024** [46]	United States of America Cross-sectional Adults	Panel Randomly selected Representative *N* = 1,224					Asked respondents whether they had changed their meat and/or seafood consumption over the past year in order to classify them as meat reducers Asked how they would describe their consumption of each of the meat/seafood food groups (a lot less than a year ago, slightly less than a year ago, about the same as a year ago, slightly more than a year ago, a lot more than a year ago) as compared to the previous year Asked what explained their changes in consumption: animal welfare, availability, convenience, environmental sustainability, familiarity, health, price, taste, or other	67% indicated that they intentionally reduced their consumption 69% indicated that they consumed red meat slightly less or a lot less than the previous year, and 64% reported consuming less processed meat. Far fewer respondents indicated consuming less poultry (34%) or seafood (26%) as compared to the previous year Health was the most frequently identified factor influencing its reduction (64.1% red meat; 62.8% processed meat; 53.3% poultry; 55.8% seafood). Price was the second most noted reason for reducing meat consumption (31.7% red meat; 23.0% processed meat; 28.6% poultry; 25.3% seafood) Only 6% reported reducing red meat due to environmental sustainability concerns as compared to 7% for poultry and 9% for seafood
**Faber et al., 2021 [**47**]**	Denmark, Germany, Spain Cross-sectional Adults	Multiple sampling methods *N* = 872 (DK: *N* = 258, DE: *N* = 296, ES: *N* = 318)			Intentions to change food intake in the coming year. Those answering yes or maybe were asked to indicate in which types of foods they would expect an increase, decrease or no change in consumption	Overall, 16.6% have the intention to change food consumption practices. 38.8% maybe Of those, 65.1% have the intention to decrease meat consumption (Denmark 72%, Spain 67%, Germany 58%)		
** Figueiredo et al., 2021** [48]	Portugal Cross-sectional Students	Other *N* = 876			Reasons that would make respondents reduce meat consumption: environment, health, ethics 5-point Likert scale	Most students are willing to reduce meat, and mainly for environmental reasons (71%, 69% health, 40% ethics) 71% agree or strongly agree to willingness to reduce meat due to environmental reasons Female students agree to environmental reasons in a higher percentage than male students. Also, significant differences between study fields, not study degree		
** Ford et al., 2023** [49]	Australia, China, United Kingdom Cross-sectional adults	Convenience (social media) *N* = 503(Australia) *N* = 785(china) *N* = 489 (UK)			“How willing are you to reduce your meat consumption in the next year for sustainability or environmental reasons?” 7-point scale	The UK was significantly more willing to reduce meat overall (M = 4.8), compared to China (M = 4.6) and Australia (M = 3.2). Gender was the next influential factor for the UK, with females significantly more willing to reduce (M = 5.3) compared to males (M = 4.1, neutral). For UK females, lamb had the highest willingness score (M = 5.7), followed by beef and pork (M = 5.4) and chicken (M = 4.5). For UK males, 18–34-year-olds are generally more willing to reduce all meat types (M = 4.9) compared to 35–65 + year-olds (M = 3.8). For China, product type was the next influential factor, with consumers significantly more willing to reduce lamb (M = 5.2), followed by beef and pork (M = 4.4) and chicken (M = 4.2). Gender was the next influential factor, with males significantly more willing to reduce all meat types than females. For Australia, age was the next influential factor, with 35–54-year-olds significantly less willing to reduce any meat type (M = 2.4), compared to 18–34 and 55–65 + year olds (M = 3.6). For all age groups, males were the least willing to reduce all meat types. In particular, males aged 35–54 were very unwilling to reduce (M = 2.2)		
** Fresán et al., 2020** [19, 50]	United States of America, Denmark Cross-sectional Adults	Multiple sampling methods Voluntary response sampling *N* = 43 (Loma Linda) *N* = 186 (Copenhagen)	‘According to you, which of the following statements do you think suits best with how lacto-ovo-vegetarian diet is perceived in your city?’	In Copenhagen, 31% thought that a vegetarian diet is considered a lifestyle as a way to mitigate climate change and for individuals who have strong animal rights beliefs (27%)			‘Which of the following areas motivated you to start your diet? Which of the following areas motivates you to continue this diet?’ Health, weight loss, environment/climate change, animal welfare, social norm, religion, taste, saving money, political reasons, convenience, diet trend	In Loma Linda, more than half of the participants reported health as the main motivation to start following a plant-based diet. Other relevant reasons were the social norm and religion In Copenhagen, around 40% mentioned concerns about animal welfare as a motivating factor, while health and environmental/climate change issues each accounted for around 22% of the responses The major reason for maintaining the diet in Loma Linda was health (69.8%), while in Copenhagen animal welfare was the main motivation to continue the diet (40.9%), followed by the environmental/climate change response (28.5%)
** García-González et al., 2020** [51]	Spain Cross-sectional Adults	Randomly selected Representative *N* = 2,052	Knowledge about the environmental impact of different food groups and questions about food sustainability knowledge in general	Around 50% of the population think that meat and derivatives contribute positively to food sustainability. This perception is more common in women and in people over 50 years Participants attribute the main negative impact on sustainability to processed foods (87%) and processed beverages (82%) The participants perceived a sustainable diet should be ‘plenty of fresh products’, ‘respectful towards biodiversity’ and ‘rich in vegetables’; while the ‘environmental impact’ showed one of the lowest scores The scores given by women were higher than those given by men for all items				
** Ginn & Lickel, 2020** [52]	United States of America Cross-sectional Adults (meat eaters)	Study 1: voluntary response sample Study 2: convenience	Study 1: ‘Indicate the effectiveness for combatting climate change of seven strategies: eating less meat; eating more organic food; buying local/seasonal/unprocessed foods; driving less; saving energy at home; installing solar panels on your house; following a plant-based diet.’ 5-point Likert scale Study 2: Assigned to read one of three messages ‘Meat consumption is a major part of our climate footprint. Meat requires more land and water to produce than other kinds of food. By eating less meat, you can decrease your climate footprint by up to 10%!’ ‘How much do you agree with this statement?’ 5-point Likert scale	Study 1: Participants rated eating less meat as the least effective strategy, followed by eating more organic food and following a plant-based diet. Driving less, using less energy at home, and installing solar panels were rated as more effective than all other strategies Meat-eater identification (low) and environmental concern (high) were related to the perceived environmental effectiveness (higher) of meat reduction Study 2: Message acceptance was descriptively lowest for the meat reduction message (3.9), compared to the reducing fuel use (4.1) and general messages (4.2), but not statistically significant. (Eating less meat was seen as less effective than taking shorter showers and driving less.) There was an interaction between meat-eater identification (low) and environmental concern (high) on the acceptance of the message (higher)	Asked to indicate their willingness to engage in seven strategies and their intentions to change their behavior over the next 6 months: eating less meat; eating more organic food; buying local, seasonal, unprocessed foods; driving less; saving energy at home; installing solar panels on your house; following a plant-based diet 5-point Likert scale	Study 1: Eating less meat (M = approx. 3.0) and following a plant-based diet (M = approx. 2.7) were the strategies participants were least willing to change. (estimated percentages out of a figure) Participants who were weakly identified as meat-eaters and who were more concerned about the environment were more willing to reduce their meat consumption Perceived effectiveness of meat reduction as a climate change mitigation strategy was associated with a willingness to reduce meat consumption Study 2: People were more willing to take shorter showers and eat more organic food than they were willing to eat less meat or drive less. Eating less meat was descriptively the lowest in willingness to change (M = approx. 3.4). (estimated percentages out of a figure)		
**Grummon et al., 2021 [**53**]**	United States of America Cross-sectional Parents	Convenience sample *N* = 544	‘Before today, had you ever heard that eating red meat can contribute to the following harms?’ (yes/no) ‘Systematic reviews of life cycle analyses indicate that production of red meat is a major contributor to greenhouse gas emissions, which are a key driver of climate change.’ as one of those harms Questions about general perceptions that red meat is bad for the environment	51% were not aware of any of the eight environmental harms. A minority of participants reported awareness that red meat contributes to environmental harms (ranging from 13% for water shortages and deforestation to 22% for climate change) Only 20% had a general perception that red meat was bad for the environment. 30% thought that red meat was good for the environment Participants aged 26–34 years reported awareness of about 0·3 fewer harms of red meat compared with those aged 18–25 years. Participants who identified as female reported being aware of fewer harms than those who identified as male. Higher educational attainment and higher income were generally associated with being aware of more harms Usual red meat consumption was not associated with being aware of more environmental harms of red meat				
** Hagmann et al., 2019** [54]	Switzerland Cross-sectional Adults	Randomly selected Representative *N* = 4,213					Rate the strength of 12 motives for low or no meat consumption: 'I eat little or no meat because …' it is better for the environment, I want to eat in an environmentally friendly way, health, regulate weight, don’t like the taste, animal welfare, my social environment, religion … 7-point Likert scale	For vegetarians, vegans and pescatarians, ethical concerns about animal welfare and environmental friendliness are stronger reasons to avoid meat consumption than for low-meat consumers Health motives did not differ between the diet styles Top motives for vegetarians: animal welfare, environmental concerns, health Top motives for pescetarians: environmental concerns, health, animal welfare Top motives for low meat consumers: health, environmental concerns, animal welfare
** Henn et al., 2022** [20]	Denmark, Germany, Spain, Poland, United Kingdom Cross-sectional Adults	Panel incl. quota *N* = 4,322			Asked to indicate their future willingness to replace a list of products with pulses 5-point Likert scale	Respondents who did not indicate a current replacement of animal-based foods stated a relatively low willingness to change in the future (40% of the sample have a low willingness to replace) Higher willingness of Spanish respondents compared to Danish respondents	Asked about the reasons to replace meat with pulses	Beef was noted as the most frequently replaced type of food, mainly driven by arguments relating to health, environment, and sustainability, especially relevant for German and Danish consumers Principal component analysis indicated that animal-based foods were replaced with pulses due to reasons of health, environment, sustainability, and other reasons, depending on the respective cultural differences Reasons to replace animal-based products among Spanish consumers distinctly differed from other countries’ drivers
**Hielkema et al., 2021 **[55]	Denmark Cross-sectional Adults	Panel incl. quota *N* = 1,005 Representative	‘It is necessary to eat and to produce less meat to reduce climate change.’ 7-point Likert scale	42% agreed, large differences between consumers in the NO intention stage (26.4%) and the intention stage (67.0%) and meat reducers (64.0%)	Transtheoretical model (5 stages of change) Asked participants how likely it was that they would stop eating meat altogether? 7-point Likert scale	57.3% precontemplation, 8.3% contemplation, 3.6% preparation, 13.8% action, 14.8% maintenance stage Younger respondents, women and respondents living in large cities were more likely to be in the intention stage than older respondents, men and respondents living in smaller cities/countryside Awareness of the climate impact of meat as driver of meat reduction Having climate-friendliness as a food choice motive increased the likelihood of being in the meat reducer stage Consumers were not planning to stop eating meat altogether: very low average scores: contemplation (M = 2.3; SD = 1.5), preparation (M = 2.3; SD = 1.5), action (M = 2.4; SD = 1.5) and maintenance (M = 2.4; SD = 1.6)		
** Janssen et al., 2016** [56]	Germany Cross-sectional Vegans	Convenience *N* = 329					‘Please name the most important reasons why you are vegan.’ Open-ended question Possible to name up to three motives	Animal-related motives (89.7%), motives related to personal well-being and/or health (69.3%), and environment-related motives (46.8%) were named most often The vast majority of respondents (81.8%) mentioned more than one motive 12.2% of the respondents referred to animal-related motives exclusively – > those have been vegan for a longer time (6.5 years) 4.9% referred to health-related motives exclusively, and 1.2% to environment-related motives exclusively Consumers driven by animal- and environment-related motives were, on average, significantly younger than those driven by animal-related motives alone
** Junges et al., 2021** [115]	Brazil Mixed-method Adults (Gauchos)	Voluntary response sample (questionnaire was sent by e-mail and shared on social networks) *N* = 433			Participants were asks about their intention to reduce or stop the consumption of different types of meat Yes or no	38% intend to reduce meat consumption Beef/bovine meat ranked first (45%) among the types of meat that respondents want to reduce or eliminate from their consumption (pork 15%, chicken 13%)	For respondents who do not consume meat, the motivations for doing so were questioned: health, animal welfare, general quality of life and environmental concerns	The item “improving my quality of life in general” was the most cited as a motivator (29%), followed by the concern with animal welfare (26%) and health (24%) 14% mentioned environmental concern as the reason for meat reduction, of which 84% were women
**Kemper et al., 2022 [**57**]**	New Zealand Cross-sectional Adults	Panel incl. quota (for gender, age and location) *N* = 913 representative	Statements on sustainable food production beliefs: "The farming of cows is harmful to the environment.", "The farming of cows is a cause of global warming." 7-point Likert scale	Meat eaters (4.0 and 3.6) were less likely to believe that farming cows is harmful to the environment and that it is a cause of global warming than meat reducers (5.0 and 4.6) and occasional meat eaters (4.6 and 4.3)	Asked to indicate reduced consumption of beef, lamb, pork, ham and/or bacon, chicken, fish, meat-substitute products, and beans, pulses, and/or legumes. (+ option "i don't eat this") 5-point Likert scale meat attachment questionnaire (4 items) 7-point Likert scale	16.3% were meat reducers 23.8% of meat eaters were attempting to reduce their meat consumption	Asked to indicate why they were reducing their meat consumption (where applicable): health, concern for animal welfare, concern for the environment, habit, and (social) appropriateness	meat reducers: 52% cost, 36% environmental concerns, 34% health concerns, 28% animal welfare meat eaters attempting to reduce meat: 61% cost, 41% environmental concerns, 36% health concerns, 34% animal welfare Little difference between meat eaters and meat reducers regarding their reasons for meat reduction Both meat reducers and occasional meat eaters were more likely to be female Meat reducers were more likely to have a bachelor’s degree or higher
** Kerschke-Risch, 2015** [58]	Germany Cross-sectional Vegans	Snowball *N* = 852					Indicate how much influence each has had on their decision to stop eating animal products: Reports on factory farming, climate protection, health, food scandals, reports on horsemeat, rotten meat, vegan friends, organic eggs deception, religion 5-point Likert scale	The three most important motives for following a vegan diet are reports on factory farming, climate protection and health Women and men did not differ in the sequence of motives
** Kley et al., 2022** [59]	Germany Cross-sectional Adults (living in Hamburg)	Randomly selected *N* = 1,214	‘Eating less meat is better for the environment’ 5-point Likert scale	Vegetarians’ agreement that eating less meat is better for the environment is higher than for non-vegetarian’s, but not statistically significant				
** Kretschmer, 2024** [109]	Germany Experiment adults	Panel Representative Control group: *N* = 491 (total *N* = 1,472)	All participants indicated in an open question what they considered as the single most effective measure one could take to reduce carbon emissions Respondents had to rank 10 abatement actions in descending order of their reduction in carbon emissions for a typical household in Germany during the time period of one year	Open question: less/no meat was named as the most effective measure by 9% Ranking: While 12.4% assign eating no meat the first rank (actual rank: 5), almost a quarter of the control group assigns it the lowest rank, more so than for any other measure. 48.5% of the control group allocate it to the lowest three ranks. Among all measures, respondents’ perceptions seem to be the most divided regarding the effects of meat consumption. It constitutes the by far largest misperception among all measures				
** Lacroix & Gifford, 2019** [60]	Canada Cross-sectional Adults	Panel *N* = 355	‘Reducing meat consumption is better for the environment’ 5-point Likert scale	Indicator was not analysed separately	Asked participants whether they were prepared to abstain from eating meat or fish on specific day(s) of the week 7-point Likert scale	Meat-reducers are most willing to further change their diet. They are willing to avoid meat on specific days of the week (M = 6.4) Moderate-hindrance meat eaters reported that they are somewhat willing to avoid meat on specific days of the week (M = 4.7) Strong-hindrance meat eaters reported that they are uncertain about their willingness to avoid meat on specific days of the week (M = 4.2)	Asked if they had already made conscious efforts to reduce their meat consumption, and if yes, what motivated these efforts Open-ended question	Meat reducers' most important motivator was health (66%), followed by ethics (60%) and environment (60%), financial benefits (43%), and social considerations (19%) Moderate-hindrance meat eaters: Health was the most common motivation (76%), followed by environmental (36%), financial (34%), ethical (32%), and social considerations (16%) Strong-hindrance meat eaters: Health was the most commonly cited motivation (68%), followed by financial benefits (41%), ethical (27%), environmental (27%), and social considerations (18%)
** Lentz et al., 2018** [61]	New Zealand Cross-sectional Adults	Panel *N* = 841 representative	‘Rate how you believe each of the following eating behaviors affects the environmental friendliness of your diet: buying foods with less packaging material, eating seasonal fruits and vegetables, buying locally sourced foods, avoiding foods that are transported by air, buying organic foods, eating less meat.’ 7-point Likert scale	The environmental benefit of consuming less meat was rated significantly lower than all other sustainable food behaviors Consumers believed that buying foods with less packaging had the greatest positive impact on the environment, followed by eating seasonally, buying local, avoiding air-transported foods, buying organic, and eating less meat Differences between abstainers, reducers and standards	‘On a scale from 1 to 7, how willing would you be to consider reducing your meat consumption sometime in the near future?’ ‘Specifically, in the next six months do you intend to reduce your meat consumption?’ Theory of planned behavior Meat attachment questionnaire	Willingness to reduce: M = 3.0 Intentions to reduce: M = 2.4 Attitudes and meat attachment were significant predictors of willingness and intentions to reduce meat intake, while subjective norm and perceived behavioral control had no consistent significant predictive ability	‘Please think back to when you first decided to reduce your meat consumption. How important were each of the following factors in influencing your initial decision to lower your overall meat intake?: health benefits, more environmentally friendly, animal welfare concerns, high cost of meat, taste preferences, weight control’ 7-point Likert scale	Motivations for reduction seem to shift across consumer groups, with different considerations rising and falling in importance depending on current meat consumption habits Top 3 motives of meat reducers: Health benefits (5.4), high cost of meat (5.0), environmental concerns and weight control (4.7) Top 3 motives of meat abstainers *N* = 25: Animal welfare (5.9), environmental concerns (4.4), taste (4.2) Among abstainers, the ‘weight control’ and ‘high cost of meat’ motivations are statistically lower, while the ‘animal welfare concerns’ motivation is statistically higher when compared to reducers. The ‘more environmentally friendly’ motivation is not statistically higher among abstainers when compared to reducers
** Liu et al., 2023** [62]	France, Brazil, China, Cameroon, South Africa Cross-sectional Adults	Convenience *N* = 16,803	‘To deal with the problems caused by the livestock industry, do you think reducing meat consumption is a good solution?’ 5-point Likert scale Does the livestock industry cause important environmental problems?	Women, highly educated people and vegetarians were more likely to agree that reducing meat consumption could be a good solution. Gender effect is only seen in France and Brazil Respondents from China, Cameroon and France were more likely to believe that reducing meat consumption could be a good option than respondents from Brazil and South Africa Women, highly educated people and vegetarians were more likely to perceive livestock as causing significant environmental problems. Gender effect is only seen in France and Brazil Brazilians were most convinced that the livestock industry causes serious environmental problems. In China, older respondents tended to agree more that livestock causes environmental problems, but not in France and Brazil				
** Loy et al., 2016** [110]	Germany Experimental study design Students	Convenience *N* = 60			‘I intend to eat less meat in the coming weeks’; ‘I would like to reduce my meat consumption in the coming weeks’; ‘I will eat less meat in the coming weeks’; ‘I will try to consume less meat in the coming week’ 7-point Likert scale	Before intervention: moderate intention to reduce meat consumption (M = 4.05) Before intervention: 61.7% of participants reported a moderate to strong intention to reduce meat consumption (4–7 points)		
** MacDonald et al., 2023** [63]	Canada Cross-sectional Students	Convenience *N* = 438					Rate the importance of 7 motives for reducing/avoiding meat: health benefits, environmental friendly, animal welfare, manage weight, taste is not enjoyable, cost effective, people around me don't eat meat 5-point Likert scale	Motives for meat reduction were similar in the two reducer groups (meat reducers and transitional meat reducers): Main motives meat reducers: health benefits (3.5), environmentally friendly (3.5), animal welfare (3.4) Main motives transitional meat reducers: environmentally friendly (3.6), health benefits (3.5), animal welfare (3.4) Vegetarians rated all motives higher than the other groups. Pattern was the most pronounced for environmental and animal welfare motives Main motives vegetarians: environmentally friendly and animal welfare (same, 4.2), health benefits (3.9)
** Malan et al., 2020** [111]	United States of America Experimental study design Students	Voluntary response sampling (in three universities) *N* = 176			‘In the next 6 months, please indicate your intention to: (1) Choose local/seasonal foods, (2) Limit processed/fast foods, (3) Eat meatless meals at least once/week, (4) Choose organic foods when possible, (5) Take only what you plan on eating’ 5-point Likert scale	Baseline: Eat meatless meals voted least favourite (M = 3.5) in control group, and in the middle (M = 4.2) in the intervention group		
** Malek et al., 2019** [64]	Australia Cross-sectional Adults (meat eaters)	Panel Representative *N* = 287	‘How big a part, if any, do you think each of the following activities play in the human contribution to climate change’ 5-point Likert scale	Low acknowledgment of the environmental impact of meat production 42% believe livestock farming plays role in human contribution to climate change. (4–5 scores) Committed meat eaters are less likely to believe livestock farming contributes to climate change (30%)	‘Indicate willingness to do the following in the coming weeks: ‘reduce meat consumption’, ‘follow a meat-free diet most of the time’, ‘avoid eating meat altogether’ and ‘follow a strict plant-based diet’.’ 5-point Likert scale	28% willing to reduce meat consumption, 40% unwilling, 32% neither willing nor unwilling A higher share of females was willing to reduce meat consumption 13% were willing to avoid meat consumption altogether in the coming weeks		
** Marinova & Bogueva, 2019** [65]	Australia Cross-sectional Adults (living in Sydney, Employees)	Panel *N* = 380	Three statements related to impacts of individual meat consumption on ecological, human and planetary wellbeing 5-point-Likert scale Six yes-or-no questions about awareness of negative impacts of current global level of meat consumption	41% disagree with meat consumption having a negative impact on the environment. 23% agree. 35% were unsure 19% think that livestock has no negative consequences on a global scale. 81% are aware of some negative consequences of global livestock industry	Intended dietary change (reduce meat consumption, stop eating meat, …)—> yes or no (choose one statement)	11% intend to reduce and 1% intend to eliminate their meat consumption (11% plan to increase meat consumption).55% do not intend to make any dietary change in the near future	Main reason for no meat consumption (options given)	Main reason for no meat consumption (*n* = 20): vegan/vegetarian conviction (10 people), animal welfare (5 people), religion (2 people), disgust (2 people) and environmental concerns (1 person)
** Mullee et al., 2017** [66]	Belgium Cross-sectional Adults (Flemish and Brussels residents)	Panel incl. quota Representative *N* = 2,436	‘Meat production is bad for the environment.’ ‘Cattle farming has a big impact on the climate.’ 6-point scale	About half of the respondents disagree (23.1%) or tend to disagree (25.7%) that meat production is bad for the environment The percentage of respondents who agreed that meat production is bad for the environment differed between vegetarians (92.1%), semi-vegetarians (52.1%), and omnivores (19.8%) More than one third (35.7%) of the respondents did not link cattle farming to the climate 94.7% of vegetarians, 60.4% of semi-vegetarians and 29.8% of omnivores agree that cattle farming has a big impact on the climate In general, larger proportions of women agreed with the statements that were more positive towards a vegetarian diet and lower meat consumption, compared with men				
** Neff et al., 2018** [67]	United States of America Cross-sectional Adults	Panel Representative *N* = 1,112					Asked whether any of the following reasons helped explain their changed consumption: health, animal welfare, environment, cost or other	Reasons for meat reduction: 51% cost, 50% health, 23% other, 12% environment, 12% animal welfare Reasons ‘concerns about environment’ and ‘cost’ were associated with reduced red and processed meat consumption
** Paiva et al., 2022** [68]	Portugal Cross-sectional Adults	Nationwide online survey + convenience sampling *N* = 491	‘Eat less meat is good for the environment’ 5-point Likert scale	40% agree (score 4 or 5)	‘I want to reduce meat consumption for the sake of the environment.’ 5-point Likert scale 4 N’s	30.6% are willing to reduce meat consumption due to environmental reasons. (4 or 5 points on scale)		
** Pickering et al., 2021** [69]	Canada Cross-sectional Young adults/adolescents (17–18 years)	Panel *N* = 487	Six actions that reduce greenhouse gas emissions were ranked for relative efficacy & compared with actual efficacy: have one fewer child, live car free, switch from electric car to car free, eat plant-based diet, recycle, upgrade lightbulbs	An efficacy knowledge score of all mitigation strategies was calculated	Transtheoretical model (4 stages of change) Asked to indicate their current stage for each of the following behaviors: Eat less meat; vacation locally instead of flying to destination, recycle, avoid products with excessive packaging, conserve energy in the home, conserve water, take public transportation, my first/next car will be electric instead of fossil fuel, have fewer children	38% precontemplation, 17% contemplation, 17% preparation, 28% action stage Subjective climate change knowledge, mitigation efficacy knowledge and locus of control positively influences likelihood of being in action stage of meat reduction Males are less likely to be in this action stage than women Unwillingness to perform in the future (precontemplation) was highest for have no children/one less child (40%) and eat less meat (38%). Three actions are reported by the majority of respondents (recycling, 80%; taking public transport, 57%; conserving energy in the home, 53%), while eating less meat has the lowest proportion (28%)		
** Ploll et al., 2020** [70]	Austria Cross-sectional Omnivore, conscientious omnivore (reduced meat consumption), vegetarian, vegan with Austrian citizenship	Multiple sampling methods (facebook groups, restaurants, vegetarian fairs, cooperation with one high school teacher, university email system)					Indicate the main reason for their last dietary change: choose one of the following answer categories: animal welfare, environment, health, taste, or other	Animal-related motives were identified as the main driver for a dietary change among vegan and vegetarian participants Vegans: 68.5% animal-related, 11.8% environment-related, 11.8% health, 2.4% taste, 5.5% other Vegetarians: 58.3% animal-related, 13.1% taste, 9.5% health, 7.1% environment-related, 10.7% other The motivational factors related to the environment were not considered relevant by earlier vegetarians (> 6 years of vegetarian eating behavior); instead, (dis-)taste played a bigger role. While animal-related motives are stable (57.9% & 60%), a significant shift is observed in the sample from distaste towards environmental-related motives: Early vegetarians: 0% environmental-related, 23.7% taste Later vegetarians: 13.3% environmental-related, 4.4% taste Among long-term vegans (> 3 years), animal-related concerns were clearly the strongest and most prevalent motives, whereas more recent vegans (≤ 3 years) assigned more importance to other motives: Long-term vegans: 86% animal-related, 7% environmental-relative, 1.8% health More recent vegans: 55.1% animal-related, 15.9% environmental-related, 18.8% health
** Pohjolainen et al., 2016** [71]	Finland Cross-sectional Adults	Randomly selected *N* = 1,890 Representative	‘Meat consumption should be lowered for environmental reasons.’ ‘By favouring Finnish meat, one can significantly cut the environmental effects of meat production.’ ‘Organic meat is a very environment-friendly product’ ‘Meat production strengthens climate change significantly more than plant production.’ 5-point Likert scale	‘Meat consumption should be lowered for environmental reasons.’: 26% agree, 35% neutral, 39% disagree Buying local and organic meat are thought to be more effective than meat reduction Respondents that do recognize the problem field well, more often agree with meat reduction ‘Meat production strengthens climate change significantly more than plant production’: 36% agree, 47% neutral, 17% disagree Females, younger and highly educated consumers are more often presented among the highly conscious				
** Rattenbury & Ruby 2023** [72]	Australia Cross-sectional Adults	Convenience (facebook) *N* = 740	29 items (15 barriers and 14 benefits) of participants' beliefs about vegetarian diet: "reduce greenhouse gas emissions", "help the environment" 5-point Likert scale Participants indicated how much they believed each of seven actions would help reduce global warming on a scale from 0 (no impact) to 10 (massive impact)	71% agree that a vegetarian diet would help reduce greenhouse gas emissions 74% agree that it would help the environment Participants viewed eating less meat as the least effective action (M = 5.5) against global warming, followed by stopping eating meat (M = 6.0)	They then indicated how willing they would be to undertake the action on a scale ranging from 0 (not at all willing) to 10 (completely willing)	People were least willing to stop eating meat (M = 5.4). Eating less meat was ranked in the middle (M = 7.1) Perceived efficacy of reducing meat intake for environmental sustainability significantly positively correlated with willingness to do so		
** Realini et al., 2023** [73]	New Zealand Cross-sectional Adults	Panel incl. quota for gender *N* = 1,061					‘How important are the following factors in motivating you to cut down on your regular meat consumption?’ Environmental concerns, reduced carbon emissions, affordability, health, weight loss, animal welfare, religion	People lowered meat consumption because of affordability (80%) and health concerns (75%) 50% thought environmental concerns were important
** Reuzé et al., 2022** [74]	France Cross-sectional Adults (non-vegetarians)	Other *N* = 25,393					Rate 12 motives: taste, health, environment, animal protection … 5-point Likert scale If agreed: ‘and it encourages me to reduce my meat consumption’ 5-point Likert scale	Over 80% declare environment as important motive to reduce meat consumption Among motives that were frequently felt important, three were frequently declared as having induced a reduction of meat consumption (> 80%): ‘healthier’, ‘better for the physical environment to limit meat’ and ‘good to vary both diet and protein sources’ Older participants were less likely to declare environment as having induced a reduction of meat consumption Highly educated individuals were more likely to declare environment as having induced a reduction of meat consumption
** Röös et al., 2022** [75]	Sweden Cross-sectional Adults	Panel incl. quota *N* = 2,497	‘Do you think that the following food has a large or small impact on the environment?’ 5-point scale	77% indicated that meat has a (rather) large negative impact on the environment (score 4 or 5)	Asked whether the respondent planned to decrease, maintain or increase their consumption of meat over the coming 2–3 years	Female, young, those in a relationship and highly educated consumers more likely to state an intention to decrease meat consumption Consumers’ view on the environmental impact of meat was the main determinant of intention to change meat consumption		
** Roozen & Raedts, 2022** [76]	Belgium Cross-sectional Adults	Convenience and snowball sampling *N* = 203	'Meat deserves to be labelled environmentally friendly.' 'Purchasing meat is a good environmental choice.' 'A person who cares about the environment would be likely to buy meat.' 'Meat is environmentally friendly and green.' 7-point Likert scale	Indicators were not analysed separately	Meat attachment questionnaire 4 N’s	In the ‘meat attachment’ model only the factor ‘dependence’ is a significant predictor for the willingness to reduce meat consumption In the 4 Ns' model, the willingness to reduce meat consumption is predicted by the belief that eating meat is not necessary and by the belief that eating meat is not normal Age, educational level and the financial situation did not significantly influence the willingness to reduce meat consumption		
** Seffen & Dohle, 2023** [77]	Germany Cross-sectional adults	Panel Representative *N* = 1,093	18 behavioral beliefs Environment 4 items, example: "Reducing my meat consumption would make me contribute to climate change mitigation." 7-point Likert scale	Environment: M = 4.69 It is worth noting that none of the beliefs referring to the environment and animal welfare showed a significant impact on attitude	Intention (3 items): I plan to reduce my meat consumption within the next four weeks How likely is it that you will reduce your meat consumption within the next four weeks? How big is your intention to reduce your meat consumption within the next 4 weeks? Willingness (3 items): In general, I can imagine reducing my meat consumption I would like to reduce my meat consumption sometime in the future How strong is your general willingness to reduce your meat consumption? 7-point Likert scale	While the general willingness to reduce meat consumption was rather high (M = 4.68), the specific intention to do so within the next 4 weeks was rather low (M = 3.54) Females showed higher levels of intention and willingness to reduce meat consumption than males		
** Siegrist et al., 2015** [78]	Switzerland Longitudinal Swiss population	Randomly selected *N* = 2,600	Participants were asked how they perceived the environmental benefits of six ecological consumption patterns: Avoid food products with excessive packaging, Buy regional foods, Buy regional foods, Eat only seasonal fruits and vegetables, Buy organic food, Eat less meat (maximum of twice per week) 6-point scale	Participants evaluated the eating less meat behavior as substantially more beneficial for the environment in 2014 (4.2) compared with 2010 (3.9), but the environmental impact of reducing meat consumption is still underestimated and rated the least beneficial in both years Females and higher-educated participants perceived reducing meat consumption as more beneficial to the environment compared with males and less-educated participants				
** Siegrist et al., 2019** [79]	Switzerland Cross-sectional French and German speaking population of Switzerland	Randomly selected *N* = 5,586	‘The production of food may have a negative impact on the environment. How do you assess this for the following foods?’ 7-point Likert scale	Imported vegetables flown in by plane were perceived as having the highest environmental impact (6.0). The second highest value was observed for conventionally produced meat. Interestingly, soy-based meat substitutes had a mean value (4.7) that was similar to that of conventionally produced meat (4.8). In contrast, organically produced meat was viewed as having much less environmental impact compared with conventionally produced meat or meat substitutes The perceived environmental impact of meat was negatively correlated with meat consumption				
** Slotnick et al., 2022** [80]	United States of America Cross-sectional Students	Convenience *N* = 721 (California) *N* = 568 (Michigan)	‘For each of the following lifestyle changes, let us know whether you think this is an effective way of combatting climate change’: Eat local/seasonal foods; Eat less meat; Drive less; Eat organic foods; Use less plastic packaging; Save energy at home; Recycle. Scale 1–4	76% (California; score 3.2) and 81% (Michigan; score 3.3) agreed to eating less meat as being effective in combatting climate change (score 3 or 4) Using less plastic was rated as most effective, followed by saving energy at home and driving less. Eating less meat fell in the middle in perceived effectiveness Women more highly rated the effectiveness of all climate change mitigation behaviors compared to men Students with a higher parental income more highly ranked eating less meat as an effective way of combatting climate change, when compared with students of lower-income parents Perceptions of meat’s climate impact were associated with lower frequency of red meat intake				
** Slotnick et al., 2024** [81]	United States of America Cross-sectional Adults (with lower incomes)	Panel Representative *N* = 1,798	“For each of the lifestyle changes, indicate whether you think this is an effective way of combatting climate change” (in random order): “eat local, seasonal foods,” “eat less meat,” “drive less,” “eat organic foods,” “use less plastic,” “save energy at home,” and “recycle.” scale from 1–4	Eating less meat was ranked as the lowest (M = 2.4) compared with all other climate-mitigation behaviors (all, P <.0001) 11% chose don't know Recycling was rated by participants as the most effective behavior (M = 3.4), followed by “use less plastic” and “save energy at home” (M = 3.3)				
**Stea et al., 2018 [**112**]**	Canada Experimental design Adults (meat eaters)	Other *N* = 593 representative	Asked to indicate from a list of possible environmental impacts which ones they believed to be associated with red meat consumption (yes or no): global warming, species loss, deforestation, natural resource depletion, natural disasters, water contamination, green house gas emissions, acid rain, climate change, soil contamination, environmental degradation, habitat loss, none, other	Awareness of the environmental impacts from red meat production was generally low Before message exposure: ‘water contamination’ was selected the most frequently (36%), followed by ‘greenhouse gases’ and ‘climate change’				
**Szczebyło et al., 2022** [82]	Poland Cross-sectional Adults (Millennials working in cities)	Panel incl. quota *N* = 317			Transtheoretical model (4 stages of change) ‘Indicate your willingness to limit your consumption of meat on a scale from 1 to 5.’ Meat attachment questionnaire	41% precontemplation, 27% contemplation, 23% preparation, 9% action 6% of the people in stage 1 want to reduce the amount of meat they eat (4 or 5 points on Likert scale). 51% of people in stage 2, 63% in stage 3 and 34% in stage 4 No relationship was found between phases of meat reduction and gender		
** Trewern et al., 2022** [117]	United Kingdom Mixed-method Adults	Convenience (online advert on retailer’s website) *N* = 92			‘How likely are you to eat less meat in the future?’ 5-point Likert scale	Pre-intervention: 29% intend to reduce meat consumption (very likely – 5 points)	‘If you plan to eat less meat in future or already don’t eat meat, please rank your motivation in order of importance to you?’ (health, environment, animal welfare, cost/saving money, social influence)	Pre-intervention: primary motivations to reduce meat consumption were to improve health (60%, *n* = 38), reduce impact on the environment (21%, *n* = 13) and concerns about animal welfare (15%, *n* = 9)
** Turnes et al., 2023** [83]	Portugal Cross-sectional Adults (living in Lisbon)	Other *N* = 197			/	67.8% are willing to reduce meat consumption due to environmental impact Being a less frequent meat consumer (< 1 time per day) is associated with a willingness 4 times higher due to environmental reasons Women were more likely to be willing to reduce meat consumption due to environmental impact		
** van den Berg et al., 2022** [84]	The Netherlands Cross-sectional Young adults	Panel *N* = 1,670 Representative			Respondents who reported to consume meat were asked to indicate whether they had the intention to reduce their meat consumption during the next five years. (yes or no) COM-B Model: 45 statements on psychological and physical capability, physical and social opportunity, and reflective and automatic motivation for reducing meat consumption 5-point Likert scale	Half of the respondents (50%) who consumed meat at least sometimes had the intention to reduce their meat consumption during the next five years, whereas 34% of the respondents did not intend to do so The intention to reduce meat consumption was comparable between males and females A trend was observed with higher frequency of meat consumption towards lower agreement to the statements in favour of reducing meat consumption	‘The environment plays an important role in my decision whether to eat meat.’	Young adults frequently reported life course transitions, especially those related to moving house, to have decreased their meat consumption The most frequently provided reasons for a decrease in meat consumption after life course transitions were independence (*n* = 88), limited meat consumption of people in the social environment (*n* = 85), saving money (*n* = 43), and environment, animal welfare, and health (*n* = 40)
** Verain & Dagevos, 2022** [86]	The Netherlands Cross-sectional Adults	Panel incl. quota (for gender, age and education level) *N* = 713 Representative	‘The consumption of meat is harmful to nature and the environment.’ ‘Eating less meat is better for the environment.’ 7-point Likert scale	Indicators were not analysed separately			Asked whether they reduced their meat consumption in the past, and if so, for what reasons (13 motives given) 7-point Likert scale	Women are more likely to be meat abstainers and meat reducers than men Meat abstainers are more often highly educated than avid meat eaters Meat abstainers valuing animal welfare significantly higher than committed meat reducers and avid meat eaters Animal friendliness is the most important motive for meat abstainers to have stopped eating meat. In addition, “makes me feel good,” environmental friendliness, health, and naturalness are important motives for stopping Like for meat abstainers, animal welfare is the number one motive for committed meat reducers to have reduced their meat consumption, followed by environmental friendliness and healthiness The small number of avid meat eaters that has reduced indicate healthiness as the most important reason, followed by affordability and sensory appeal
** Verain et al., 2021** [85]	The Netherlands Cross-sectional Adults	Panel incl. quota (for gender, age and education level) *N* = 1,979 Representative			Meat consumption increase/decrease in the past year and intended change in meat consumption in the next year 7-point scale (1 = much less meat; 7 = much more meat) Meat attachment questionnaire 4 N's	Reported intentions to further decrease meat consumption in the coming year (mean = 3.6) Compulsive meat eaters (4.1), meat lovers (3.9), unconscious flexitarians (3.6), potential flexitarians (3.3), conscious flexitarians (2.8) The large majority of conscious flexitarians was female (87%) The distribution in education level did not differ across the flexitarian segments	Asked whether they reduced their meat consumption in the past, and if so, for what reasons (13 motives given) 7-point Likert scale	Top three motives: compulsive meat eater: affordable (4.7), sensory appeal (4.6), safe (4.4) meat lovers: Affordable and healthy (4.6), safe (4.4) unconscious flexitarians: healthy (5.1), affordable (4.9), sensory appeal (4.8) potential flexitarians: makes me feel good (5.7), animal friendly and environmental friendly (5.6) conscious flexitarians: animal friendly (6.1), healthy (6.0), environmental friendly (5.9) Self-reported decrease in meat consumption in the past year: mean = 3.5 (1 = much less meat; 7 = much more meat)
** Verain et al., 2024** [87]	The Netherlands Cross-sectional Adults	Panel with quota Representative *N* = 1,982			Transtheoretical model of health behavior change: Would you describe yourself as a flexitarian (definition given)? ‘No, and I do not intend to become a flexitarian in the next 6 months’ (pre-contemplation), ‘No, but I intend to become a flexitarian in the next 6 months’ (contemplation), ‘No, but I intend to become a flexitarian in the next 30 days’ (preparation), ‘Yes, I am a flexitarian, but not for more than 6 months yet’ (action) or ‘Yes, I am a flexitarian and I have been a flexitarian for the past 6 months or longer’ (maintenance) Willingness to gradual change was measured with a slider, on which respondents could indicate the percentage that they were willing to reduce their meat consumption meat attachment questionnaire	54.7% of the respondents did not intend to reduce their meat consumption (precontemplation stage), 10.2% of the respondents intended to reduce their meat consumption, but did not take action yet (contemplation and preparation stages) and the remaining 32.1% of the participants already started to actively reduce their meat consumption (action and maintenance) On average participants are willing to reduce with 41%, but large differences exist between participants. About a quarter of the participants is only willing to reduce their meat consumption by 10% or less, whereas also about a quarter of the participants is willing to reduce their meat consumption with 70% or more In the no intention group, the willingness to reduce is lowest, with a mean reduction of 24%. The intention group is willing to reduce with about 56%. The performing group is most willing to reduce, with about 62%		
** Wolstenholme et al., 2021** [88]	United Kingdom, Italy Cross-sectional Students (meat eaters)	- *N* = 320 UK sample *N* = 304 Italian sample			Transtheoretical model (4 stages of change) Theory of planned behavior INT1: "I intend to reduce my weekly red/processed meat consumption”, INT2: "I plan to reduce my weekly red/processed meat consumption”, INT3: “I will reduce my weekly red/processed meat consumption" 7-point Likert scale - > summarized to one variable ‘intention’	Italian sample: 17% precontemplation, 5% contemplation, 9% preparation, 17.5% action (estimated percentages out of a figure) UK sample: 21% precontemplation, 8% contemplation, 15% preparation, 8% action (estimated percentages out of a figure) Significantly more UK participants were in the preparation stage as compared to Italian participants, while significantly more Italian participants were in the action stage as compared to UK participants Participants did not intend to reduce their red and processed meat consumption, but were also not reluctant to do so (3.9 in both samples [UK and Italian]) Intention to reduce red and processed meat was significantly predicted by attitude, subjective norm and perceived behavioral control Intention to reduce red and processed meat was not predicted by meat-eater identity. But meat-eater identity had a significant negative effect on attitude and perceived behavioral control—> indirect effect on intention to reduce		
** Wynes et al., 2020** [89]	North America Cross-sectional Adults and students	- *N* = 551 (adults) *N* = 414 (students)	Participants were asked to categorize 15 actions as low (< 1% of a person’s carbon footprint), medium (1–5%), or high impact (> 5%) Open-ended question concerning the most effective action they could take to reduce greenhouse gases that contribute to climate change	Switching from driving an SUV to public transit received the highest average ranking (60% ranked it as high) Eating a vegan diet is ranked high by 23% (same as switching from plastic to canvas bags). 48% rank it as low Open-ended question: Most frequent response type was actions related to reduced driving. Eat less meat is on 7th place (of 25)				
**Qualitative studies**								
**Author, publication year**	**Country, Study design, Population under study**	**Sampling method, sample size**	**Indicator measured** **RQ1: Awareness**	**Key findings** **RQ1: Awareness**	**Indicator measured** **RQ2: Willingness to reduce**	**Key findings** **RQ2: Willingness to reduce**	**Indicator measured** **RQ3: Motives for reduction**	**Key findings** **RQ3: Motives for reduction**
** Austgulen et al., 2018** [113]	Australia Mixed-method (focus groups) Adults	Panel *N* = 24			One theme included a discussion of willingness to change to less or no meat	None of the participants said they would like to become a vegetarian or make large cutbacks in their meat consumption Young women were most positive about changing their consumption Participants expressed the need for serious proof in order to be persuaded to change their food habits in order to reduce climate change, or wished that climate-friendly initiatives would be introduced in silence		
** Campbell-Arvai, 2015** [114]	United States of America Mixed-methods (focus groups) Students	Multiple sampling methods (Recruitment letters were mailed to a random sample of students. Two focus groups with students enrolled in a freshman environmental programme.) *N* = 40	In the final stage of the discussion, the facilitator asked all participants about the connections they made (if any) between their own food choices, food production practices and the health of the environment, as well as what specific choices or actions they thought could be taken to minimize potential negative environmental outcomes	In each focus group, at least one student mentioned that for them, there was no connection between their food choices and environmental sustainability. Instead, they spoke of other behaviors that they believed would have more of an impact, i.e. taking shorter showers, switching off lights and computers or driving a fuel-efficient car In all focus groups, the issue of food waste had a strong connection to environmental sustainability and the health of the environment			Semi-structured interview protocol	Only students recruited from the environmental programme indicated that they had adopted a vegetarian diet because of the environmental impacts of raising food animals
** Cleland et al., 2025** [90]	Scotland Focus groups adults	Multiple repeating the study conducted by Macdiarmid et al. (2016) *N* = 60	“Do you think that the type of food we choose has an impact on the environment? If yes, what do you think those foods are?” “Are some foods better or worse than others for the environment? Which ones?” “Some people think what we eat is contributing to climate change. What do you think of it?” “Some people think that eating less meat would be good for the environment and climate change. What do you think about this argument?”	Participants exhibited varying levels of awareness regarding the environmental impact of meat consumption. This suggests that awareness of the impact of livestock and farming on climate change has increased and there is a broader knowledge than in 2013/14 focus groups “the potential impact of that [reducing meat] on climate is enormous because meat rearing is the biggest single thing that contributes to that dietary impact on climate change.” Like the previous study, the relative importance of meat compared to other sectors (e.g., air travel) was raised, with the suggestion that meat consumption was of lesser environmental concern than these other contributors. There was a belief that the impact of meat was overemphasised However, views diverged between sociodemographic groups. Few participants in urban, high deprivation groups acknowledged that meat consumption has an impact on climate change	Would you be willing to reduce the amount of meat you eat for the sake of the environment and climate change?	Participants tended to possess a greater willingness to reduce meat consumption than in the 2013/14 study Clear disparity between sociodemographic groups: Many LD participants expressed willingness to reduce their meat consumption, or reported having already done so, thereby feeling that there was no need to reduce it further. In contrast, reluctance to reduce meat consumption was common among participants in rural HD groups, with some expressing disagreement about the environmental benefits of meat reduction and emphasising the necessity of meat in a 'balanced' diet Even those who are aware of the need to change often believe that other actions, such as reducing plastic waste from packaging or choosing more local options may be the most impactful actions they can take, with this belief remaining constant in the decade since the previous study		Some cited environmental concerns as prompts for change In the LD groups, reductions in meat consumption were attributed to health concerns and animal welfare considerations. In HD groups the high cost of meat dominated discussions: “I think I'm doing it [reducing meat] already but not necessarily for the environment. If somebody wants me to pay £5 for a steak, I'm sorry.”
** Collier et al., 2021** [18]	Sweden Focus groups	Other (Online recruitment system) *N* = 33	Semi-structured discussions One of the core topics was reducing meat consumption (general thoughts around this—why or why not, potential benefits, health and climate perspectives, ways to reduce meat in the diet, and their desire to eat more vegetarian generally)	Many participants were aware that meat consumption negatively impacts the environment Some degree of scepticism of this was expressed by a number of participants. This subtheme was especially prevalent among the pre-contemplation groups. One participant commented that they believed that meat consumption cannot only be bad for the environment, because although cows produce methane gas, their manure helps vegetation grow which binds carbon Some participants believed that there was not enough information available regarding meat and climate change, and that the available information just repeats that meat is bad for the environment without providing convincing evidence or explaining why Some participants seemed sceptical to the idea that changing their own behavior could have much impact, since they had already reduced their meat consumption or do not perceive themselves as eating much meat in the first place It seemed that lack of awareness of, or scepticism towards, the impact of meat on the environment promoted other behaviors such as sourcing locally produced meat rather than reducing meat consumption. Rather these seemed to fuel general Scepticism to the need to reduce or substitute meat, since these other behaviors were perceived as sufficient in themselves			Reducing meat consumption as a core topic: general thoughts around this—why or why not, potential benefits, health and climate perspectives, ways to reduce meat in the diet	Some had already reduced their meat consumption, because of the negative impacts of meat consumption on the environment There were participants (mostly in the action/maintenance stage group) who had already reduced their meat consumption for health reasons For some, maintaining good health was a reason for continuing to eat meat as they currently do, some advocated for more meat in the diet for improved health, while for others health was a motivation for reducing, or attempting to reduce, their meat consumption
** Collier et al., 2022** [91]	Sweden Focus groups Adults intending to reduce meat consumption or who were vegetarian/vegan and living in Gothenburg	Voluntary response sampling (through an online recruitment system) *N* = 39	Extent to which participants agree that they care about what they eat in terms of themselves, the environment, and/or animals	Redirections [in conversations], typically to health or practical concerns seemed to serve as a strategy to explain why they struggled with changing their behavior, despite their awareness of the negative aspects of meat consumption	-	Many meat-eating participants indicated that they were strongly considering eating less or rejecting meat entirely, but that they simultaneously did not feel able, willing, or ready to do so Participants were not willing to completely cut out meat, instead preferring to at most reduce or eliminate red meat while retaining other meat and seafood products in their diets	-	Animal welfare seemed to be a strong motivator among vegetarians/vegans, with other aspects such as the environment and health perspectives being perceived as additional benefits “No, it was the animal industry and it, like in the beginning, then there’s been more and more discussion about the environment in the past fifteen years so now it’s because of the environment.” (F36-a) Decision to reduce meat is: “...not only about the environment, it’s simply myself, like egoistically motivated, that I want to be healthier.” (F52-c)
** Fenzl et al., 2022** [92]	Austria, Australia, Indonesia Interviews Students (international students)	Convenience *N* = 25	The authors were interested in the attitudes toward meat consumption and the thoughts with regard to food choices and the environment	Eight participants (32%) believed that there is a direct connection between meat consumption and climate change, also indicating, that less meat consumption would be better for the environment Some participants described not knowing enough about the topic or not knowing anything about the interconnection of food choices and the environment (24%, six interviewees)				
** Ford et al., 2023** [93]	United Kingdom Focus groups Young adults	Convenience *N* = 38	‘Some people think 'what we eat is contributing to climate change'. Do you agree with this statement?’ ‘Some people believe 'eating less meat and dairy would be good for the environment'. Do you agree with this statement?’	Overall, participants were very knowledgeable regarding the environmental impact of the livestock industry and consumption of meat, perceiving it to play a contributing role in climate change However, notable knowledge gaps were also observed, specifically relating to uncertainty around making sustainable food choices Reducing meat mentioned with medium frequency, e.g.: ‘I feel like eating sustainably is mostly about cutting out meat, so red meats.’	‘How willing would you be to reduce your meat consumption for the environment’s sake?’	86% were slightly, moderate or extremely willing to reduce meat consumption Despite consciously recognising the sustainability benefits of reducing meat and dairy, some participants were still unwilling to reduce highlighting an awareness behavior gap	‘What influenced you to change your consumption habits?’ Key themes were presented using the COM-B model	Main reasons: moving away from family and limited food budget Environmental and sustainability reasons were mentioned as additional motives. Also, consumer trends and health benefits were mentioned by fewer participants
**Hazley et al., 2024** [94]	Ireland Interviews Adults	Convenience *N* = 20	Semi-structured questionnaire (open-ended questions) Could you describe what you think an environmentally sustainable diet is? Do you ever consider a foods environmental impact when buying food?	Most expressed a degree of uncertainty when they described what an environmentally sustainable diet meant to them Some admitted that they had never considered the environmental impact of food until being asked in the interview Most participants did not mention meat when discussing a sustainable diet Those who saw meat reduction as environmentally beneficial often spoke of it as an “ideal” rather than something they planned to do themselves Why reducing meat was more sustainable was not well understood. Few seemed aware of the direct emissions produced by livestock. Some felt animal products were less sustainable as they required more processing or had more plastic packaging than plant-based foods			Have you made any changes to your diet to make it more sustainable? Why?	Even vegetarian participants felt the environmental benefits were secondary to the ethical and health reasons that initially drove them to avoid meat
** Hoek et al., 2017** [95]	Australia Interviews Adults	Quota sampling from panel *N* = 29	Interviewer asked what the man/woman in the picture was thinking if they would shop for “an environmentally friendly meal” 4 behaviors suggested by professionals: eating less animal- and more plant-derived foods ‘What words come to mind when you read this’, ‘How would you explain that to someone else’, ‘Have you ever tried to do this/would you be interested in this’	Low knowledge and awareness of impact on environment Environmentally friendly was associated with credence attributes “organic” and “free range”, and more related to packaging. Some participants confused this with ethical aspects, which are not necessarily related to environmental impact. Compared to health, the relationship between food and the environment is rarely considered by consumers ‘Doing stuff for environment I think more of saving water and electricity around the house and things like that.’	4 behaviors suggested by professionals: eating less animal- and more plant-derived foods ‘What words come to mind when you read this’, ‘How would you explain that to someone else’, ‘Have you ever tried to do this/would you be interested in this’	Participants had the most positive attitude and highest motivation for eating less processed and packaged foods, followed by reducing food waste and overconsumption There was a predominantly negative attitude towards, and low motivation for, eating less animal-derived products and more plant-based foods Disapproval/irritated with suggestion of meat reduction It was mostly associated with a complete vegetarian/vegan lifestyle, by which participants could not identify themselves with, and did not consider in between options	-	"It's never been the sole reason to do something with food because of the environment"
**Junges et al.,** **2021** [115]	Brazil Mixed-method (interviews) Adults (Gauchos)	- *N* = 14			semi-structured script Asked about the interest in reducing the consumption of red and white meat	The type of meat that the interviewees mentioned seeking to reduce was beef (but not for the reason of climate protection)		
** Kemper, 2020** [21]	New Zealand Focus groups Adults (trying to reduce their meat consumption and living in two major New Zealand cities)	Voluntary response *N* = 36					-	Significant differences in motivations for meat reduction between young adults, families, and retirees, with health, environmental and cost important factors: Young adults either reduced their consumption due to environmental or health concerns. However, the push to reduce meat was also a social pressure through friends, flatmates, and social media Families reduced their meat consumption for either child allergies (health, such as eczema) or cost, and to a much lesser extent environmental impact For retirees the reason for meat reduction was their lack of appetite and enjoyment of meat. Participants regularly mentioned switching from red meat to fish and chicken None wanted to eliminate meat completely
** Khara et al., 2021** [96]	Australia Interviews Adults (living in Sydney)	Voluntary response (via facebook and university career websites) *N* = 22			-	Some participants expressed interest in cutting back further on meat consumption and adopting more plant-based foods but they also identified several challenges	-	They have reduced consumption of red meat in favour of fish and chicken for health reasons Others reported purchasing local meat as they considered it more environmentally friendly Rising environmental and health consciousness, and concerns for animal welfare have also contributed to dietary changes "I try now to eat less protein [reference to red meat]…for health reasons, and I guess, in some ways, I guess, it’s better for the environment…The most important reason is health reasons. Secondary would be the environment.."
** Loiselle et al., 2024** [97]	Canada Explorative -	Convenience *N* = 55	"What are or would be the advantages/disadvantages of reducing your red meat consumption over the next three months?” open-ended question	17.2% mention reduction of red meat consumption as being good for the environment	theory of planned behavior			
**Macdiarmid et al., 2015 [**98**]**	Scotland Focus groups and interviews Adults	Several methods: randomly selected and voluntary response sampling *N* = 83 (focus groups) *N* = 4 (interviews)	‘Some people think what we eat is contributing to climate change.’ ‘Some people think that eating less meat would be good for the environment.’	Some participants were unaware or had not previously thought of food having an environmental impact or that it was contributing to climate change Lack of awareness of the association between meat consumption and climate change Discussants typically described food packaging, food waste, transportation of food and production and processing of food in relation to the environmental impact of food Some believed that compared with other behaviors meat consumption was trivial Other human activities such as transportation, pollution from industry and powerstations were often regarded as more environmentally damaging than food production or eating meat Perceptions of personal meat consumption playing a minimal role in a global context of climate change	‘Would you be willing to reduce the amount of meat you eat for the sake of the environment?’	Resistance to the idea of reducing personal meat consumption This finding emerged across all socio-economic groups, and there did not appear to be obvious differences in the responses by sex or location (i.e. rural or urban) of the participants Those who claimed to have already reduced their meat intakes (particularly red meat) or only ate small quantities of meat in general believed that they did not need to reduce it further ‘I am aware that ruminants cause a problem with methane, that wouldn't stop me eating meat.’ ‘I probably won't eat less meat. I'm aware of the environment, I take other steps, fine I do my bit, recycling, driving less but I probably wouldn't change my diet.’	-	Reasons given for cutting down meat included health concerns, food scares (e.g. horse meat scandal), the high cost of meat, living with a partner who was vegetarian or changing dietary habits with ageing These were views expressed by both men and women and across all socio-economic groups No mentioning of climate protection as a motive
** Mann et al., 2018** [99]	Australia Interviews Adults	Voluntary response and convenience sampling *N* = 24	‘On a scale from 1 to 10, how important are the following things to you when you shop for food and drink at the supermarket?’ ‘On a scale of 1–10, how much impact do you think the following eating behaviors/characteristics have on the environment?’ 9-food behaviors and characteristics	The majority of participants had never come across information about a sustainable eating pattern Participants displayed limited knowledge about an environmentally sustainable eating pattern Participants underrated the environmental impact of the farming, processing and packaging of food products A very small number of respondents believed that food did not have any impact on the environment Eating fresh food products in season, eating lots of fish and eating lots of meat were considered to have a moderate environmental impact Eating organic, locally produced, natural foods, following a vegetarian diet or eating less meat were each mentioned by a smaller number of participants When asked about the environmental impact of meat and dairy, many participants stated that these products may have some environmental impact; however, these products were also considered to be healthy	‘On a scale of 1–10, how willing are you to undertake the following eating behaviors?’ 7-food behaviors	Participants were more willing to undertake sustainable food behaviors if there were further perceived benefits other than just helping the environment Many participants were willing to follow a sustainable eating pattern; however, the specific behaviors they were willing to undertake varied. Even though many participants were willing to follow a plant-based eating pattern with limited meat consumption, a few participants were adamantly unwilling as meat was seen as a necessary part of the diet The sustainable food behaviors that participants were most willing to undertake were the behaviors that could be considered the easiest to undertake or that require the smallest shift in their current eating pattern	‘Do you ever try to follow an environmentally sustainable eating pattern for environmental reasons?’	Overwhelmingly, health was the main reason consumers followed or were willing to follow a sustainable eating pattern “I do follow this type of eating pattern […] however, it is more for personal reasons that I do this. Environment yes is just an additional factor I see it as.”
** McBey et al., 2019** [100]	Scottland Focus groups Adults	Convenience - (11 focus groups)	-	Knowledge of the link between meat consumption and environmental degradation was limited The student group seemed most informed on the various problems associated with livestock production	Topic guide: thoughts on dietary change as one topic	‘It's food, I don't care what the environmental impact is, it's food. If you want to be good to the environment we can make savings in other areas, not food. We need it to survive.’		
** Mohr & Schlich, 2016** [116]	Germany Mixed-method Adults (from Rhineland-Palatinate	Multiple sampling methods (quota and snowball) *N* = 1,040			‘What reasons influence your actual willingness for reducing the consumption of meat and sausage goods?’ open-ended question (N = 380 participants answered)	40% say because meat is a non-essential component of the diet Health is clearly a driving factor for reducing meat consumption (33.6%); animal protection (14.8%), protection of resources (6.9%) Noone mentionend climate protection Altruistic factors or abstract aspects of food consumption (e.g. climate protection) are generally of less significance		
** North et al., 2021** [101]	Australia Inductive, qualitative approach Adults	Snowball (via social media) *N* = 701					‘What are the reasons you follow this diet?’ [vegan or vegetarian] Open-ended question	Vegans: 62% animal welfare, 54% environment, 51% health Vegetarians: 43% animal welfare, 43% environment, 40% health The top four motivations for vegetarians were the same as for vegans. However, vegetarians also had more of the other motivations co-occurring
**O’Keefe et al., 2015 [**102**]**	United Kingdom Focus groups Consumers	Voluntary response sampling *N* = 40	Warm-up discussion about sustainability	None of the groups mentioned climate change directly in this prompted discussion on sustainability Only a minority discussed the emissions implications (i.e. environmental impact) of eating meat	Participants were asked how they viewed a 20% and a 70% reduction in meat consumption and whether it was achievable	The majority of participants stated they were comfortable with the suggestion of a 20 per cent reduction in meat consumption ‘I wouldn’t find it a huge issue because I’ve already gone through that eating meat almost every night to a couple of times a week. It’s not really that hard.’ When asked about a 70 per cent reduction in meat, this was considered too drastic a shift from current practices, leading participants to suggest clearer dietary guidance from government sources would be needed to adopt such changes. A 70 per cent meat reduction was frequently referred to as a “vegetarian” diet by participants Parents reported they would be happy to reduce meat consumption for themselves but not for their children, due to their perceptions of the role of meat in satisfying nutritional needs Females considered men in their families would find both a 20 and 70 per cent reduction in meat consumption problematic. Males, however, expressed similar levels of personal willingness to reduce meat consumption	-	Climate change and sustainability do not influence purchasing decisions Animal welfare issues were often mentioned alongside price and quality in discussions around respondents’ current reasons for reducing meat consumption The absence of meanings related to sustainability when participants discussed their current food habits suggests that presenting change exclusively in terms of environmental benefits may have limited effect
** Ortiz et al., 2022** [103]	Argentina Focus groups Adults (citizens of Cordoba)	Non-probabilistic *N* = 59	-	The role of meat consumption for sustainability is almost unnoticed by most participants The overconsumption of meat was recognised only among the youngest and some of those in the environmentalist group, and exclusively by females				
**Randers et al., 2020 **[104]	Denmark Interviews -	Convenience (interviewer’s own social network) *N* = 13	semi-structured interviews	Citation of a ‘ethical foodie’: ‘Our consumption reflects the production system, so if we stop eating meat, then we will not contribute to that industry, which hopefully will be reduced emitting less CO2.’			‘What are the main reasons that you eat/do not eat meat?’	The three pragmatic idealists (vegans/vegetarians) expressed that being exposed to information about animal suffering and environmental degradation caused by the food industry had fueled their idealism through increased awareness and changed beliefs regarding animal welfare and environmentalism Three ethical foodies had a low consumption of meat due to environmental concerns, concerns about animal welfare in conventional meat production, and health concerns. Taste was also commonly referred to as a central motive Healthy hedoists: Ethical reasons behind food choices were of minor relevance or not mentioned at all in this group
**Tucker et al., 2018** [105]	New Zealand Focus groups New Zealanders from 16–66 years	Convenience *N* = 69	‘In what ways might meat consumption be reduced in Aotearoa New Zealand?’ One option: more meat-less meals	Overall, participants’ positions on reducing meat consumption were positive: 38 people stated support for this, with just two opposing			-	Three main drivers for reducing meat consumption by frequency of participant comment were: economic, health/nutrition and aesthetics/taste Environmental motivations when thinking about meat production and consumption are in simple terms, sparse
**Whittall et al., 2022 **[106]	United Kingdom Interviews Young adults	Convenience *N* = 21	‘Can you tell me about what you understand by, or what do you think a sustainable diet is?’ ‘What impact do you think a sustainable diet would have on the planet?’	Participants were able to define sustainable eating in a manner that was more or less consistent with the definition by the FAO (e.g. plant-based diets), and could identify sustainable actions that they could undertake to make their diets more sustainable (e.g. meat reduction) But there was also a lot of uncertainty over definition, actions, or lacking confidence/uncertainty: ‘I would say a sustainable diet would be stuff that we grow, so foods like potatoes and veg. I think a sustainable diet would be one day you go vegetarian), but I don’t really know […] what a sustainable […] diet would be, if I’m honest.’	‘Would you like to adopt a more sustainable diet?’ ‘If you wanted to, would you know how to make your diet more sustainable?’ ‘How do you think you could make changes to your diet?’ ‘Would you be willing to make these changes?’	Participants wanted to do more and were willing to, but they didn’t know what they should be doing, and consequently weren’t doing anything There was a clear interest in small changes; participants were resistant to making large changes to their diets		
** Wolstenholme et al., 2024** [107]	United Kingdom, Brazil Interviews UK: adults Brazil: students	Multiple UK: *N* = 22 Brazil: *N* = 41	awareness of the environmental impacts of eating meat (interview guideline not given)	Knowledge of the environmental impacts of livestock production varied substantially within each country In the UK sample, awareness tended to be particularly low among meat-eating compared to vegetarian/vegan participants. In Brazil, mixed levels of awareness were shown across meat-eating and vegetarian participants Some participants did not think that reducing their meat consumption would have much of a positive impact on the environment and were sceptical about the effects of individual change on environmental issues	4 N’s		motivations for avoiding meat and other animal products	The main motivation found to encourage vegetarians and vegans was animal welfare Health did not appear to motivate vegetarian/vegan diets initially, however vegetarian/vegan participants noted the health benefits of not eating meat Some meat-eating participants had reduced their meat consumption for environmental reasons. In these cases, participants had specifically reduced their beef consumption, acknowledging the greater environmental impacts of beef Participants tended to become informed about the environmental consequences of meat production only after they had already adopted a vegan/vegetarian diet for another reason, generally relating to animal welfare. Many participants stating that environmental reasons were an important motivation for continuing to exclude meat from their diet

#### Quantitative studies

Awareness of the link between meat consumption and the potential to mitigate climate change was most often measured by asking about the level of agreement with meat-specific mitigation potential statements [39–41, 44, 52, 55, 59–62, 68, 71, 72, 76–78, 80, 81, 113, 114] or by asking about level of agreement with statements about the environmental impact of meat [36, 37, 51, 53, 57, 62, 64–66, 71, 75, 79].

Statements about mitigation potential were often as follows: ‘Eating less meat is better for the environment’ [59] or ‘To deal with the problems caused by the livestock industry, do you think reducing meat consumption is a good solution?’ [62]. Participants were asked to rate their level of agreement, in most cases on a scale from one to five. In some studies, agreement on meat-specific mitigation strategies was compared to agreements on other mitigation strategies, such as ‘buy local, seasonal, unprocessed foods’, ‘buy/eat more organic foods’, ‘drive less’, ‘recycle’ and ‘save energy at home’ [41, 52, 61, 69, 72, 78, 80, 81, 89, 109, 113, 114].

In four out of the nine articles reporting the percentage of participants who agreed with the mitigation potential of meat reduction, only a minority of participants (less than 30%) agreed with such statements [39, 41, 71, 114]. Only in two studies, one on a convenience sample of students (2022) and one on adults (2024), did more than half agree that eating less (red) meat was better for the environment and an effective way to combat climate change [40, 80]. When comparing the perceived mitigation potential of meat reduction with other mitigation strategies, meat reduction is highly underestimated and often ranked as the least effective [41, 52, 61, 71, 72, 78, 81, 109, 113, 114]. Only in two non-representative studies (2020 and 2022) eating less meat was ranked somewhere in the middle [80, 89]. In a longitudinal study, participants rated eating less meat as substantially more beneficial for the environment in 2014 than in 2010 [78]. However, the environmental benefit of reducing meat consumption still has a low relevance rating and was rated the least beneficial. The perceived effectiveness of eating less meat appears to be negatively associated with individual meat-eating status (eight out of eight studies) [41, 44, 52, 55, 59, 61, 62, 80]. Five out of six studies that analyzed data by gender found that women were more likely to agree with the mitigation potential of eating less meat [40, 41, 62, 78, 80].

Questions regarding the awareness of the environmental impact of meat for example asked: ‘Do you think that the following food has a large or small impact on the environment? ‘ [75] or ‘To what extent do the following activities contribute to climate change?' [36] (‘The production of meat and dairy products, which we eat and drink’ was one of those). Again, the level of agreement was rated (the level of scales varied between studies, from four to ten).

In articles reporting the percentage of participants who agreed with the environmental impact of meat consumption, the percentages of individuals who were aware range from 20% [53] to 77% [75]. In 12 out of 15 studies, general awareness of the environmental impact is low (less than half of the participants acknowledge the environmental impact) [37, 50, 51, 53, 64–66, 71, 112] or was rated as low relevance [61, 79, 89]. Again, the perceived environmental impact of meat appears to be negatively associated with current meat consumption (five out of six studies) [57, 62, 64, 66, 79].

#### Qualitative studies

Only a small number of qualitative studies provide their interview guidelines, so we cannot always replicate how awareness of the link between meat consumption and climate change was measured.

The results of qualitative research are consistent with the quantitative research, suggesting that there is little or some awareness of the link between meat consumption and climate change, but scepticism remains and other behaviors are perceived to play a greater role, e.g., ‘Doing stuff for environment I think more of saving water and electricity around the house and things like that.’ (Australian study, 2017 [95]). However, one recent study from Scotland (2025) [90] tried to replicate a study from 2016 [98] and suggests that awareness regarding the environmental impact of meat has increased.

#### Research question 2: What is the current state of knowledge regarding individuals’ willingness to reduce meat consumption in order to mitigate climate change?

Forty-eight included articles provide information on the second research question, including 31 quantitative [20, 22, 35, 36, 38, 40, 41, 44, 45, 47–49, 52, 55, 57, 60, 61, 64, 65, 68, 69, 72, 75–77, 82–85, 87, 88], ten qualitative [90, 91, 93, 95, 96, 98–100, 102, 106], three intervention [108, 110, 111] and four mixed method studies [113, 115–117] (see Table 2).

#### Quantitative studies

Six studies measure willingness to reduce meat consumption using the transtheoretical model of health behavior change by Prochaska and Velicer [118], where the authors chose to use four [35, 69, 82, 88] or five [55, 87] stages of change: precontemplation, contemplation, preparation, action, and in two cases an additional stage for maintenance. People in the contemplation or preparation stage are counted as individuals willing to reduce meat consumption, while those in the action and maintenance stage have already reduced their meat intake. The percentages of people in the contemplation stage range from 6 to 27%, in the preparation stage from 4 to 45%, and in the action stage from 9 to 35%.

The wording of the questions used in the studies varies widely from willingness and intentions to planning, preparing and wanting to reduce meat consumption. Most studies used 5-point Likert scales, but also other scales or no scales but simple agreement (yes or no) were used to indicate willingness to reduce meat consumption. Some studies include a specific time frame in their question, such as ‘over the next 6 months’, others include a non-specific time frame, such as ‘in the near future’, and some studies do not mention a time component at all. Most studies report percentages of people willing or unwilling to reduce meat consumption. Others report averages of agreement on their 5-point or 7-point Likert scale.

In three studies [44, 61, 88] the Theory of Planned Behavior by Ajzen [119], in six studies [57, 61, 76, 82, 85, 86] the meat attachment questionnaire by Graça et al. [120], and in four studies [68, 76, 85, 86] the 4N’s by Piazza et al. [121] were used to explain individual willingness/intention to reduce meat consumption.

Due to the wide variety of methods used for describing willingness/intentions to reduce meat consumption it is difficult to summarize the results of the included studies. Several studies have shown that willingness to reduce meat consumption is associated with female gender (9 out of 11 studies) [22, 41, 48, 55, 64, 69, 75, 77, 83], current meat consumption of participants (inversely associated; 8 out of 11 studies) [22, 41, 44, 52, 60, 83–85], perceived effectiveness of meat reduction for climate mitigation (4 out of 4 studies) [41, 52, 69, 72] and awareness of the climate impact of meat (3 out of 3 studies) [55, 69, 75].

Six studies compared willingness to reduce meat consumption to willingness to implement other behavioral changes to mitigate climate change or protect the environment, such as eating more organic food, saving energy at home, or driving less: all studies found willingness to reduce meat consumption to be among the lowest rated [41, 52, 69, 72, 108, 111].

Four studies asked about the likelihood of stopping eating meat altogether: In three studies, consumers were very unlikely to stop eating meat altogether [55, 65, 72]. In the third study (representative Australian study from 2019), 15% were willing to avoid eating meat in the coming weeks [64].

#### Qualitative studies

One finding from qualitative studies is that participants often confuse a reduction in meat consumption with complete abstinence from meat, which they refuse to implement, while preferring to make smaller changes to their diet [91, 95, 99, 102, 106, 113]. For example, a study by O’Keefe et al. [102] showed that the majority of the study sample was comfortable with a 20 percent reduction in meat consumption, but rejected a proposed 70 percent reduction. Similar to quantitative studies, other mitigation measures were also discussed in qualitative studies, and participants often preferred them over the option of eating less meat [90, 95, 98–100]. Individuals’ willingness to reduce meat consumption varies between the studies, with one study of young adults (UK, 2023) [93] showing a strikingly high percentage (86%) of participants being willing to reduce their meat consumption for the sake of the environment. In one study (Germany, 2016), participants were asked in an open-ended question about the reasons influencing their actual willingness to reduce meat consumption [116], and no one mentioned climate protection.

#### Research question 3: What is the current evidence on individuals who have reduced their meat consumption for climate change mitigation reasons?

Thirty-eight included articles address the third research question: 20 quantitative studies [20, 22, 36, 45, 46, 50, 54, 56–58, 60, 61, 63, 65, 67, 70, 73, 74, 84, 85], 14 qualitative studies [18, 21, 90, 91, 93–96, 98, 99, 102, 104, 105, 107], and four mixed method studies [113–115, 117] (see Table 2). Two studies conducted in Germany in 2015 and 2016 included only vegans [56, 58]. Five other studies compared motives between vegetarians and vegans [70] or between vegetarians and “meat reducers” [22, 54, 61, 63].

#### Quantitative studies

Motives for reducing or eliminating meat from the diet were often asked by giving multiple-choice options and asking participants to rate their importance (13 out of 22 studies), such as health benefits, animal welfare, environmental concerns, religious reasons, taste or saving money [46, 50, 54, 57, 58, 61, 63, 67, 73, 74, 85, 115, 117]. Five studies specifically asked about the main reason(s) for eating little or no meat: three gave multiple options [22, 65, 70] and two studies used an open-ended question [56, 60]. Two studies asked directly whether (red) meat consumption had been reduced for environmental reasons [36, 113] and one study asked about reasons for replacing meat with pulses [20]. One study used the Vegetarian Eating Motives Inventory [45].

When meat reducers (excluding meat abstainers) were asked about their motives in multiple-choice questions, environmental concern was usually not ranked highest, but it was usually in the top three (11 out of 14 studies) [20, 50, 54, 57, 61, 63, 67, 73, 74, 85, 117], most often after health and animal welfare. When asked an open-ended question, the rankings are similar, with health being the most important motivator for meat reducers [60] and animal welfare being the most important for meat abstainers [56]. An Italian study from 2023 asked directly whether red meat consumption had been reduced for environmental reasons and found that about half of the participants answered this question in the affirmative, and 7% had even stopped eating red meat altogether for environmental reasons [36]. A Norwegian study from 2018 found that 14% of respondents reported reducing their overall meat consumption for environmental reasons [113].

One of the studies among vegans showed that the most important motives for following a vegan diet were reports on factory farming, climate protection, and health [58], while the second study found that vegans were mainly motivated by animal-related motives (90%), motives related to personal well-being and/or health (69%), and less frequently by environment-related motives (47%) [56]. While the majority of respondents (82%) cited more than one motive, 12% exclusively referred to animal-related motives—these have been vegan for a longer time (at least 6.5 years). Vegans driven by animal- and environment-related motives were on average significantly younger than those driven by animal-related motives alone. Similarly, an Austrian study from 2020 found that environmental motives were less relevant for long-term vegetarians (> 6 years of vegetarian eating behavior) and vegans (> 3 years), than for newer vegetarians and vegans [70].

In three out of four studies, animal welfare was significantly more important for vegetarians/vegans than for meat reducers [54, 61, 63]. Two studies found that environmental protection was a higher motivator for vegetarians than non-vegetarians [54, 63], while two others found no significant difference [22, 61].

#### Qualitative studies

Again, only a minority of qualitative studies provide their interview guidelines, so it’s often difficult to understand how reasons for meat reduction were asked.

Similar to quantitative findings, multiple motivations were reported in qualitative studies. Health and animal welfare were common motivators for reducing meat consumption [18, 91, 94, 96, 98, 99, 102, 105, 107]. Environmental protection was mentioned by some participants in more recent studies [18, 21, 96, 101, 104], e.g. specifically among vegetarians (Danish study, 2020) [104]/vegans (Australian study, 2021) [101] and young adults (New Zealand study, 2020) [21], and otherwise mostly seen as an additional benefit [91, 93–96, 99]. However, some studies [91, 107] suggest that environment is an important motivator for continuing to exclude meat from the diet, e.g., a quote from a Swedish study in 2022: “No, it was the animal industry and it, like in the beginning, then there’s been more and more discussion about the environment in the past fifteen years so now it’s because of the environment” [91].

## Discussion

### Summary of key findings

This scoping review synthesizes evidence from 93 studies on individuals' perspectives on reducing meat consumption to mitigate climate change. The majority of studies were published in 2019 or later, were conducted in Europe among adults, and used a quantitative study design. Most studies were carried out in non-representative and small populations, limiting the generalizability of the results to national and international levels. There were large differences between studies in the way the questions and answer options for a topic were formulated. This makes it difficult to compare the results. The results suggest that individual awareness of the mitigation potential of reducing meat consumption is low. The perceived environmental impact of meat and the perceived effectiveness of reducing meat consumption appear to be negatively related to current amount of meat consumption. In addition, several studies have shown that the willingness to eat less meat is positively associated with female gender and negatively associated with the current level of meat consumption. When people are asked about their motivations for eating less meat, climate protection is not the main motive, but it is usually one of the three most frequently cited motives; the most frequently cited motives are health benefits and animal welfare.

### Strengths and limitations

A strength of this scoping review is that we used the Preferred Reporting Items for Systematic Reviews and Meta-Analyses extension for Scoping Reviews (PRISMA-ScR) guidelines to ensure a systematic approach to searching, screening and reporting. We also considered that both climate change and meat consumption are studied in different disciplines, so the databases searched cover health, medical, environmental and social sciences. In addition, we focused on recent evidence (as of 2015), as awareness and attitudes may have changed significantly in recent years, as attention to the topic has increased with the Paris Agreement, a legally binding international treaty on climate change [2], which was issued and signed by many countries in December 2015.

This scoping review also has several limitations. First, grey literature was not included, such as national polls or consumer market research not published in peer-reviewed journals, even though these data may shed light on meat consumption habits and behavior change intentions of populations. We only included peer-reviewed articles to ensure a certain standard of good scientific practice for the evidence summarized. In addition, we did not perform a risk of bias assessment for each included study. However, this is in line with the PRISMA-ScR extensions for scoping reviews. A risk of bias assessment is applicable to systematic reviews of interventions, where scoping reviews are “generally conducted to provide an overview of the existing evidence regardless of methodological quality or risk of bias” [33]. Furthermore, because we included a variety of study types, it would not have been feasible to use a single tool to assess risk of bias. Finally, we did not define the term “meat reducer” in our eligibility criteria, so being a meat reducer may have different meanings, just as the term “flexitarian diet” is not clearly and/or consistently defined in research.

### Implications for policy and practice

The studies reviewed use a wide variety of study designs and questions to assess the willingness and motivation to reduce meat consumption and awareness of its role in climate change. The generalizability of the results is hampered by the fact that many studies have rather small sample sizes and are mostly conducted in Western countries, although dietary behavior depends on many aspects such as culture, social norms and food availability. Generalizability and comparability across societies could be improved by conducting research in nationwide samples from more countries and using a standardized, validated instrument or greater harmonization of questions between studies. Better and more detailed documentation of the interview process in qualitative studies may also contribute to comparability. Overcoming these methodological limitations in future research is important for advancing the understanding of how to effectively promote a more plant-based diet in populations.

A number of studies included in this scoping review used the Transtheoretical Model of Health Behavior Change (TTM) by Prochaska and Velicer [116] or the Theory of Planned Behavior (TPB) by Ajzen [117] to explain willingness to reduce meat consumption. The TTM is a good tool for assessing people’s readiness to adopt a particular health behavior, in this case reducing meat consumption. The stages of change can be used to design interventions, which are more likely to be fruitful, if they meet people where they are, rather than a ‘one fits all’ approach. The theory of planned behavior has been used to predict a variety of (health) behaviors and is good at explaining people’s intentions to do something [122]. However, it has its limitations in explaining actual behavior change, as it is well known that people do not always act on their intentions. Another more recently developed framework for studying behavior is the COM-B model [123]. It considers both barriers and facilitators while incorporating individual capabilities, opportunities, and motivations for a particular behavior. The COM-B framework may also be used as a tool for investigating meat consumption behavior and could be helpful in implementing policies that make it easier to choose meat-free meals.

This scoping review identified a gap in research on this topic for children and adolescents. While children do not usually prepare their own meals, they still have to make food choices on a regular basis, e.g. in the school canteen. Generating evidence in younger age groups on the awareness of the benefits of meat reduction may be informative, as dietary habits are often established in childhood and strengthened in adolescence. In addition, there are hints that adolescents’ preferences may even make family meals more sustainable [124]. Young adulthood is a critical period to re-evaluate one’s eating habits, often in combination with moving away from home. At that point, it is crucial that young adults can make fully informed decisions about their food consumption. Another research gap was found for regions other than Western countries. The vast majority of included studies were conducted in Europe, North America, and Oceania. Studies from low- and middle- income countries are missing. Since some low- and middle- income countries are trying to increase their meat availability, and especially in larger cities, to adapt more and more to a Western diet, it would be very interesting to monitor awareness of and attitudes to meat consumption in these countries in further research.

It is also important to monitor people’s knowledge and behavior regarding meat consumption and its environmental impact over time. The dynamics of media coverage on climate change and its drivers can be expected to raise the population-wide awareness in the present and the near future, and may motivate people to change their diets to a more plant-based diet. The food industry and retailers have a growing interest in expanding markets for alternative proteins and are starting to promote plant-based meat alternatives; marketing campaigns can contribute to raising awareness [125].

Improving awareness and knowledge of the environmental impact of meat consumption may lead to increased willingness and ultimately to a reduction in meat consumption. Awareness-raising campaigns and health education could be provided in school canteens, day-care institutions, workplaces, cooking classes, supermarkets, etc. The benefits of reducing meat consumption for health, climate change, animal welfare and others should be emphasized, while explaining that a total abstinence is not necessary.

National food-based dietary guidelines for healthy eating should be re-evaluated considering also the environmental impact of food production, as some countries, such as Germany [126] or Denmark [127], have already done. Increasing the visibility and variety of vegetarian dishes in food environments and increasing the availability and affordability of meat alternatives can make it easier for consumers to switch to a more plant-based diet [17]. Policies can help make the (environmentally) healthier choice the easier choice by providing tax reductions or subsidies for vegetables/fruits and tax increases for meat and dairy products, which can lead to health, environmental and economic benefits [128–130]. According to several systematic reviews, such adjustments in taxation are likely to be effective in improving diets [131–133]. Among food providers, nudging the more environmentally friendly and healthier dishes, for example by placing it at the top of the menu or by labeling it as the ‘dish of the day’, is a very simple but effective measure that can be implemented by canteens/restaurants to increase the consumption of (environmentally) healthier foods [17, 134]. It may also be effective to reduce the portion size of meat and substituting it with plant-based components [17].

## Conclusions

This scoping review synthesizes the findings of 93 studies on individuals' perspectives on reducing meat consumption as a climate change mitigation strategy. The majority of the studies were conducted in Europe and used quantitative, cross-sectional designs with non-representative study samples. Regarding awareness, the review highlights that while some people are aware of the environmental impact of meat consumption, overall awareness remains low and many underestimate its role in climate change mitigation. Awareness of the environmental impact is negatively associated with current meat consumption. When it comes to willingness to reduce meat consumption, factors such as gender, awareness of climate impact, and perceived effectiveness of meat reduction play an important role. However, other environmental behaviors, such as reducing car use or saving energy, are often prioritized over dietary changes. In terms of motivations, health and animal welfare concerns often take precedence, with environmental motivations being secondary for most participants. However, some recent studies suggest that climate-related motivations may be gaining in importance.

To improve comparability across studies, future research could benefit from harmonization and validation of questionnaires. Using these standardized instruments in population-wide studies would increase the generalizability of the results. As media and political attention to climate change mitigation increases, it will be interesting to monitor changes in individuals’ perspectives on meat reduction in relation to the three research questions.

To facilitate a shift away from high meat diets, awareness campaigns should target different settings, including schools, workplaces, and supermarkets. Educating consumers about the health, environmental, and ethical benefits of reducing meat consumption—and emphasizing that complete abstinence is not necessary—could help overcome resistance. In addition, structural changes, such as improving the availability and affordability of vegetarian alternatives, for example in canteens, will be key to making plant-based diets more accessible to a wider population.

## Data Availability

All data analysed in this scoping review are included in this article.
